# Multi-locus phylogeny of lethal amanitas: Implications for species diversity and historical biogeography

**DOI:** 10.1186/1471-2148-14-143

**Published:** 2014-06-21

**Authors:** Qing Cai, Rodham E Tulloss, Li P Tang, Bau Tolgor, Ping Zhang, Zuo H Chen, Zhu L Yang

**Affiliations:** 1 Key Laboratory for Plant Diversity and Biogeography of East Asia, Kunming Institute of Botany, Chinese Academy of Sciences, Kunming, Yunnan 650201, China; 2 Herbarium Rooseveltensis Amanitarum, P. O. Box 57, Roosevelt, New Jersey 08555-0057, USA; 3 School of Pharmacology, Kunming Medical University, Kunming, Yunnan 650500, China; 4 Institute of Mycology, Jilin Agricultural University, Changchun, Jilin 130118, China; 5 College of Life Science, Hunan Normal University, Changsha, Hunan 410081, China; 6 University of Chinese Academy of Sciences, Beijing 100039, China

**Keywords:** Amanita, Biogeography, Lethal substances, Phylogenetic species, Molecular clock, Synapomorphy

## Abstract

**Background:**

Lethal amanitas (*Amanita* section *Phalloideae*) are a group of wild, fatal mushrooms causing many poisoning cases worldwide. However, the diversity and evolutionary history of these lethal mushrooms remain poorly known due to the limited sampling and insufficient gene fragments employed for phylogenetic analyses. In this study, five gene loci (nrLSU, ITS, *rpb2*, *ef1*-α and β-*tubulin*) with a widely geographic sampling from East and South Asia, Europe, North and Central America, South Africa and Australia were analysed with maximum-likelihood, maximum-parsimony and Bayesian inference methods. Biochemical analyses were also conducted with intention to detect amatoxins and phalloidin in 14 representative samples.

**Result:**

Lethal amanitas were robustly supported to be a monophyletic group after excluding five species that were provisionally defined as lethal amanitas based on morphological studies. In lethal amanitas, 28 phylogenetic species were recognised by integrating molecular phylogenetic analyses with morphological studies, and 14 of them represented putatively new species. The biochemical analyses indicated a single origin of cyclic peptide toxins (amatoxins and phalloidin) within *Amanita* and suggested that this kind of toxins seemed to be a synapomorphy of lethal amanitas. Molecular dating through BEAST and biogeographic analyses with LAGRANGE and RASP indicated that lethal amanitas most likely originated in the Palaeotropics with the present crown group dated around 64.92 Mya in the early Paleocene, and the East Asia–eastern North America or Eurasia–North America–Central America disjunct distribution patterns were primarily established during the middle Oligocene to Miocene.

**Conclusion:**

The cryptic diversity found in this study indicates that the species diversity of lethal amanitas is strongly underestimated under the current taxonomy. The intercontinental sister species or sister groups relationships among East Asia and eastern North America or Eurasia–North America–Central America within lethal amanitas are best explained by the diversification model of Palaeotropical origin, dispersal via the Bering Land Bridge, followed by regional vicariance speciation resulting from climate change during the middle Oligocene to the present. These findings indicate the importance of both dispersal and vicariance in shaping the intercontinental distributions of these ectomycorrhizal fungi.

## Background

*Amanita* Pers. (Agaricales, Basidiomycota) is a cosmopolitan genus comprising about 500 described and accepted species [1-3]. This genus is one of the most known fungal genera because it comprises both deadly poisonous species, e.g., *A. phalloides* (Fr. : Fr.) Link and valued edible species, e.g., *A. caesarea* (Scop. : Fr.) Pers. The genus *Amanita* also plays important roles in forest ecosystems, as a large majority of the species are known to be ectomycorrhizal fungi (ECF) [4]. They are primarily associated with Araucariaceae, Betulaceae, Casuarinaceae, Dipterocarpaceae, Fabaceae, Fagaceae, Myrtaceae, Nothofagaceae, Pinaceae and Polygonaceae [2-8]. In the traditional classifications based on morphological and anatomical characters, *Amanita* was often split into two subgenera, *Amanita* and *Lepidella* (J.-E. Gilbert) Veselý [9,10], comprising seven sections *Amanita*, *Caesareae* Singer, *Vaginatae* (Fr.) Quél., *Amidella* (J.-E. Gilbert) Konrad & Maubl., *Lepidella*, *Phalloideae* (Fr.) Quél. and *Validae* (Fr.) Quél [8]. Molecular phylogenetic studies on *Amanita* have generally supported these morphological-taxonomic treatments, except for the monophyly of the section *Lepidella*[11-14].

Lethal amanitas are a group of deadly poisonous mushrooms included in the section *Phalloideae*, and all of them are ECF. In recent decades, mushroom poisoning cases caused by lethal amanitas have been frequently reported, which account for over 90% of all fatal mushroom poisonings worldwide [15-19]. These amanitas share several common phenotypic features, including a non-appendiculate pileus, the persistent presence of an annulus, a bulbous stipe base with a limbate volva and amyloid basidiospores [3,8-10]. These features make lethal amanitas relatively easy to distinguish from other taxa of *Amanita*. The presence of lethal substances in these amanitas also make them distinct from other amanitas. The lethal constituents are a kind of cyclic peptide toxins which can be divided into three primary groups: amatoxins, phallotoxins and virotoxins [20].

According to the literature and herbarium surveys, lethal amanitas comprise ca. 50 species worldwide and the majority of them are centred in the Northern Hemisphere (particularly East Asia and North America) in which ca. 37 species have been recorded [3,5,6,9,21-28]. However, despite recent reports of species with both morphological and molecular evidences, the classifications of most amanitas are based exclusively on morphological studies. Thus, taxonomic positions of these species remain largely unverified because identifying different species of lethal amanitas based on morphological and anatomical evidences is often difficult and controversial due to morphological similarities and the paucity of characteristic traits. Furthermore, previous molecular phylogenetic studies of lethal amanitas have either been based on limited sampling or that the employed gene markers were insufficient to resolve the phylogeny [12-14,27,29-34]. Therefore, the true diversity and evolutionary history of lethal amanitas remains largely unknown.

In this study, with a broader sampling, multiple and independent DNA gene fragment analysis in combination with biochemical and morphological analyses, our study intends to (1) test the monophyly of lethal amanitas and assess their phylogenetic position; (2) generate a globally representative molecular phylogeny of lethal amanitas; and (3) reconstruct a biogeographic diversification pattern of lethal amanitas, with an emphasis on their diversification in the Northern Hemisphere.

## Methods

### Taxon sampling

One hundred fifteen samples from East and South Asia, Europe, North and Central Americas, Africa and Australia were included in this study, representing 33 species of putative lethal amanitas. The samples sequenced in this study were deposited in the Cryptogamic Herbarium of Kunming Institute of Botany, Chinese Academy of Sciences (HKAS), Herbarium of Mycology, Jilin Agricultural University (HMJAU), Mycological Herbarium of Guangdong Institute of Microbiology (GDGM) and the Private Herbarium Rooseveltensis Amanitarum (RET). Each specimen’s scientific name, GenBank accession number and other relevant information included in this study are listed in Additional file 1: Table S1. Forty-eight internal transcribed spacer (ITS) sequences of the nuclear ribosomal RNA were retrieved from GenBank (NCBI; http://blast.ncbi.nlm.nih.gov/; Additional file 2: Table S2). Four species of *A*. subgen. *Amanita* were selected as outgroups based on previous studies [11,14,32].

### DNA extraction and sequencing

Genomic DNA was extracted from fruiting bodies dried in silica gel or from herbarium specimens using the modified CTAB method [35]. Five DNA gene fragments were analysed, including those coding for the second-largest subunit of RNA polymerase II (*rpb2*), translation elongation factor subunit 1α (*ef1*-α) and beta-tubulin (β-*tubulin*), along with two non-protein coding regions, ITS and nrLSU. Primer pairs ITS1/ITS4 or ITS1F/ITS4 [36,37], LROR/LR5 [38] and 983 F/1567R [39] were used to amplify ITS, nrLSU and *ef1*-α, respectively. For *rpb2* and β-*tubulin*, initial attempts to amplify using previously published primers designed for fungi [34,40] resulted in weak or non-specific amplification. To improve the success rate, new primers were designed based on our newly obtained sequences using the online program Primer3 [41]. For β-*tubulin*, we designed a new primer pair (Am-β-*tubulin* F: 5′-AAG CGG AGC RGG TAA CAA YTG G-3′; Am-β*-tubulin* R: 5′-ACR AGY TGG TGR ACR GAG AGY G-3′), that covers the positions 5–26 and 454–475 of *A. pantherina* (DC. : Fr.) Krombh. [GenBank: AB095881], and 5–26, 449–470 of *A. muscaria* (L. : Fr.) Lam. [GenBank: DQ060923]. For *rpb2*, a primer pair (Am-6 F: 5′-TGG GGA ATG GTR TGY CCT GC-3′; Am-7R: 5′-CCC ATK GCT TGT TTR CCC ATG GC-3′) was designed by reducing the degeneracy of the primers used in Matheny [40].

The PCR reactions were conducted on an ABI 2720 Thermal Cycler (Applied Biosystems, Foster City, CA, USA) or an Eppendorf Master Cycler (Eppendorf, Netheler-Hinz, Hamburg, Germany), and the reactions were conducted using the following profiles: pre-denaturation at 94°C for 4 min, followed by 35 cycles of denaturation at 94°C for 40 s, annealing at 48°C–54°C for 40 s, elongation at 72°C for 60 s (β-*tubulin*), 90 s (ITS, *ef1*-α, and *rpb2*) or 150 s (nrLSU), and a final elongation at 72°C for 8 min. The PCR products were purified before sequencing using a Gel Extraction and PCR Purification Combo Kit (Spin-coloum) (Bioteke, Beijing, China). The purified products were then sequenced on an ABI-3730-XL DNA Analyser (Applied Biosystem, Foster City, CA, USA) using the same primer combinations used for the PCR. Forward and reverse sequences were assembled and edited with SeqMan (DNA STAR package; DNAStar Inc., Madison, WI, USA).

### Sequence alignment and phylogenetic analysis

We compiled two datasets, the ITS sequences matrix and a concatenated dataset (nrLSU, *rpb2*, *ef1*-α and β-*tubulin*), to investigate the phylogeny of lethal amanitas. In the combined dataset, the sequences of nrLSU, *rpb2*, *ef1*-α and β-*tubulin* were aligned initially using MUSCLE v3.8.31 [42], and then manually optimised on BioEdit v7.0.5 [43]. To test for potential conflicts among the four gene fragments, maximum likelihood analyses and Bayesian Inference were performed on each individual dataset set with the same setting as in the concatenated analysis (see below). As no conflicts were found (e.g. well-supported differences in the topology; Additional file 3: Figure S1), the four gene fragments were combined with Phyutility [44] for further phylogenetic analysis. Some ambiguously aligned regions, that were characterised by uncertain positions and the presence of introns, were excluded from subsequent analyses.

The ITS dataset was complemented by related sequences from GenBank (http://www.ncbi.nlm.nih.gov/) using the genus search tool complement in emerencia [45]. Taxa outside the section *Phalloideae* and sequences with little information due to short lengths were discarded after preliminary alignment using the program MAFFT v6.8 [46]. They were realigned using MAFFT and manually edited in 4SALE v1.5 [47]. The realigned matrix contained 134 sequences, and 114 of them were the sequences of putative lethal amanitas.

Maximum likelihood (ML) analysis was conducted on RAxML v7.2.6 [48]. To estimate the branch support with alternative methods, we performed maximum parsimony (MP) bootstrap analysis and Bayesian inference (BI) analysis with PAUP4.0b10 [49] and MrBayes V3.2 [50], respectively. For the ML and BI analyses, the optimal substitution model was determined using the Akaike Information Criterion (AIC) as implemented in MrModeltest v2.3 [51]. The selected substitution models for the five partitions were as follows: General Time Reversible + Proportion Invariant + Gamma (GTR + I + G) for nrLSU, ITS and *rpb2*, Symmetrical model (SYM) + I + G for *ef1*-α and Hasegawa-Kishino-Yano (HKY) + I + G for β-*tubulin*. In the ML analysis, the statistical supports were obtained using a rapid bootstrapping with 1000 replicates, and the other parameters used the default settings. The concatenated dataset was partitioned into four parts by sequence regions. Some of the selected models could not be implemented in RAxML, and thus the GTR + I + G model, which included all of the parameters of the selected model, was used instead. For BI analysis, the combined dataset was partitioned as in the ML analysis, and a partitioned mixed model analysis allowing model parameters to be estimated separately for each gene was applied. Bayesian tree topology and posterior probabilities (PP) were determined from two independent runs of one cold and three heated chains. Runs were performed for 50 million generations with trees sampled every 100 generations. Chain convergence was determined using Tracer v1.5 (http://tree.bio.ed.ac.uk/software/tracer/) to confirm sufficiently large ESS values (>200). Subsequently, the sampled trees were summarised after omitting the first 25% of trees as burn-in using the ‘sump’ and ‘sumt’ command implemented in MrBayes.

MP analyses were performed in PAUP [49] with a heuristic search of 1000 replicates with random stepwise addition using tree-bisection-reconnection (TBR) branch swapping and starting from trees obtained by the stepwise addition of sequences. All of the characters were equally weighted and gaps were treated as missing data. Parsimony bootstrap (PB) analyses [52] with 1000 replicates were subsequently performed using the fast bootstrap option to evaluate the robustness of the MP trees.

### Biochemical analysis

Fourteen dried specimens were selected for biochemical analyses. They represented all four sections of *A.* subgen. *Lepidella*, with an emphasis on the species which were morphologically assigned in the section *Phalloideae* but were phylogenetically clustered outside the section in our analyses. The presence of α-amanitin, β-amanitin and phalloidin (standard samples provided by Sigma Chemical Co, USA) was evaluated through high-performance liquid chromatography (HPLC) with the method of Chen et al. [53].

### Phylogenetic species determination

Molecular phylogenetic species of lethal amanitas were delimited according to two criteria. The first was the genealogical concordance phylogenetic species recognition (GCPSR) criterion [54], which has been proved to be useful in fungi and is currently the most wildly used identification method in the fungi kingdom [55-57]. Phylogenetic species were recognised as genealogically exclusive under GCPSR if they were concordantly supported by multiple independent loci. The second identification criterion was based on the ITS sequences because some of the samples from Europe, North America and South Africa lacked *rpb2*, *ef1*-α and β-*tubulin* sequences in the GenBank database. In this method, the phylogenetic species were recognised according to the inter- and intra-specific variations of the ITS sequence, using criteria established to define phylogenetic or environmental species in previous studies [58-60]. Generally speaking, the intraspecific variations of some well-studied species were first compared, and then the highest variation was defined as the threshold to delimit species within this group. In this study, *A. exitialis* Zhu L. Yang & T.H. Li, *A. fuliginea* Hongo, *A. fuligineoides* P. Zhang & Zhu L. Yang, *A. ocreata* Peck, *A. pallidorosea* P. Zhang & Zhu L. Yang, *A. phalloides*, *A. rimosa* P. Zhang & Zhu L. Yang, *A. subjunquillea* S. Imai and *A. virosa* (Fr.) Bertillon, which have been thoroughly studied using both morphological and molecular analyses [3,5,27,31], were selected to establish the conservative cutoff value. Afterwards, all of the well supported terminal branches in the phylogenetic tree inferred from the ITS sequences were treated provisionally as separate species, and then the intra- and inter-specific ITS variations within and among them were calculated and compared with the cutoff value. Any provisionally adopted species showing larger intraspecific variations than the cutoff value were further split into two or more species, and any two or more species harbouring inter-specific variations lower than the cutoff value were combined into a single species. All of the inter- and intra- specific ITS variations were calculated in MEGA v.4.0 using the Maximum Composite Likelihood model [61].

### Molecular dating analysis

Given that fossil records of fungi are limited, it has been difficult to choose a reliable calibration point to estimate the divergence time for any fungal groups. Therefore, an extensive sampling of outgroup species for which fossils were available were selected to estimate the divergence time of *Amanita*. Two primary calibration points were included in our analyses: (1) the divergence between Ascomycota and Basidiomycota, 582 Mya, by placing *Paleopyrenomycites devonicus* in the subphylum Pezizomycotina [62]; and (2) the divergence between Hymenochaetaceae and Fomitopsidaceae based on the 125 million-year-old fossil, *Quatsinoporites cranhamii*[63]. The parameter settings for the two calibrations were the same as those used in Feng et al. [59]. As the identifications of the two fossils were largely ambiguous, the estimated divergence time was constrained by the following two values. The estimated divergence time between Ascomycota and Basidiomycota is at least 400 Mya (the divergence time of *P. devonicus*). The initial diversification of *Amanita* and lethal amanitas should be close to the divergence time of their host plants suggested by the co-estimation of the fungi and the plants [64]. The calibration point by which the estimated results met the two criteria was eventually chosen for our analyses.

Three gene fragments, nrLSU, *rpb2* and *ef1*-α, were concatenated for molecular dating using the phylogenetic framework described in James et al. [65]. All of the outgroup sequences were retrieved from the nrLSU, *rpb2* and *ef1*-α alignments in the AFToL database [66] (Additional file 4: Table S3). ModelTest v2.3 was used to select the best models of evolution using the hierarchical likelihood ratio test. The origin time of *Amanita* was estimated in BEAST v.1.6.1 [67], with the molecular clock and substitutions models unlinked but trees linked for each gene partition. The BEAST input files were constructed using BEAUti (within BEAST), in which the GTR + G + I model was selected. The lognormal relaxed molecular clock model and the Yule speciation prior set were used to estimate the divergence times and the corresponding credibility intervals. The posterior distributions of parameters were obtained using MCMC analysis for 50 million generations with a burn-in percentage of 10%. The convergence of the chains was checked using Tracer v1.5. Samples from the posterior distributions were summarised on a maximum clade credibility (MCC) tree with the maximum sum of posterior probabilities on its internal nodes using TreeAnnotator v1.6.1 [67] with the posterior probabilities limit set to 0.5 to summarise the mean node heights. FigTree v1.4.0 (http:// tree. bio. ed. ac. uk/software/Figtree) was used to visualize the resulting tree and to obtain the means and 95% higher posterior densities (HPD) [67]. A 95% HPD marks the shortest interval that contains 95% of the values sampled.

We also estimated the divergence times of the main nodes in lethal amanitas using the ITS dataset, which contained one or two representatives of all of the lethal amanitas included in our analyses. The estimated crown age of lethal amanitas by the combined nrLSU, *rpb2* and *ef1*-α dataset was used as the calibration point to date the ITS phylogeny by setting the prior to a normal distribution. The other procedures were the same as those applied in the estimation using the combined dataset.

### Biogeographic analysis

The reconstruction of ancestral areas in a phylogeny is important in understanding the biogeographic diversification history of a lineage, as this makes it possible to infer the original place and dispersal routes of organisms. We employed two alternative event-based methods to infer ancestral areas: (1) a Bayesian Binary MCMC analysis as implemented in the computer software Reconstruct Ancestral States in Phylogenies (RASP) [68], and (2) a maximum likelihood-based method implemented in the computer program LAGRANGE [69]. The ancestral area analyses were conducted on the posterior distribution of the dated ITS phylogeny estimated from BEAST. In the Bayesian MCMC analyses, we chose the F81 model, allowing for different rates of change among ancestral areas. The Bayesian posterior probabilities were determined twice by running 10 chains over 100, 000 generations, saving reconstructions every 100 generations.

The program LAGRANGE was used to run the maximum likelihood analysis, with a simple model involving single rate of dispersal and extinction set as constant over time and across lineages. This program estimates not only the ancestral area for each node but also the probability of range inheritance scenarios. Those regions where the lethal amanitas were introduced with host plants were not included in our analyses. Seven areas were finally delimited: (A) East Asia, (B) Europe, (C) eastern North America, (D) western North America, (E) Central America, (F) Australia and South Africa and (G) South Asia.

## Results

### Phylogenetic analysis

**Figure 1:**
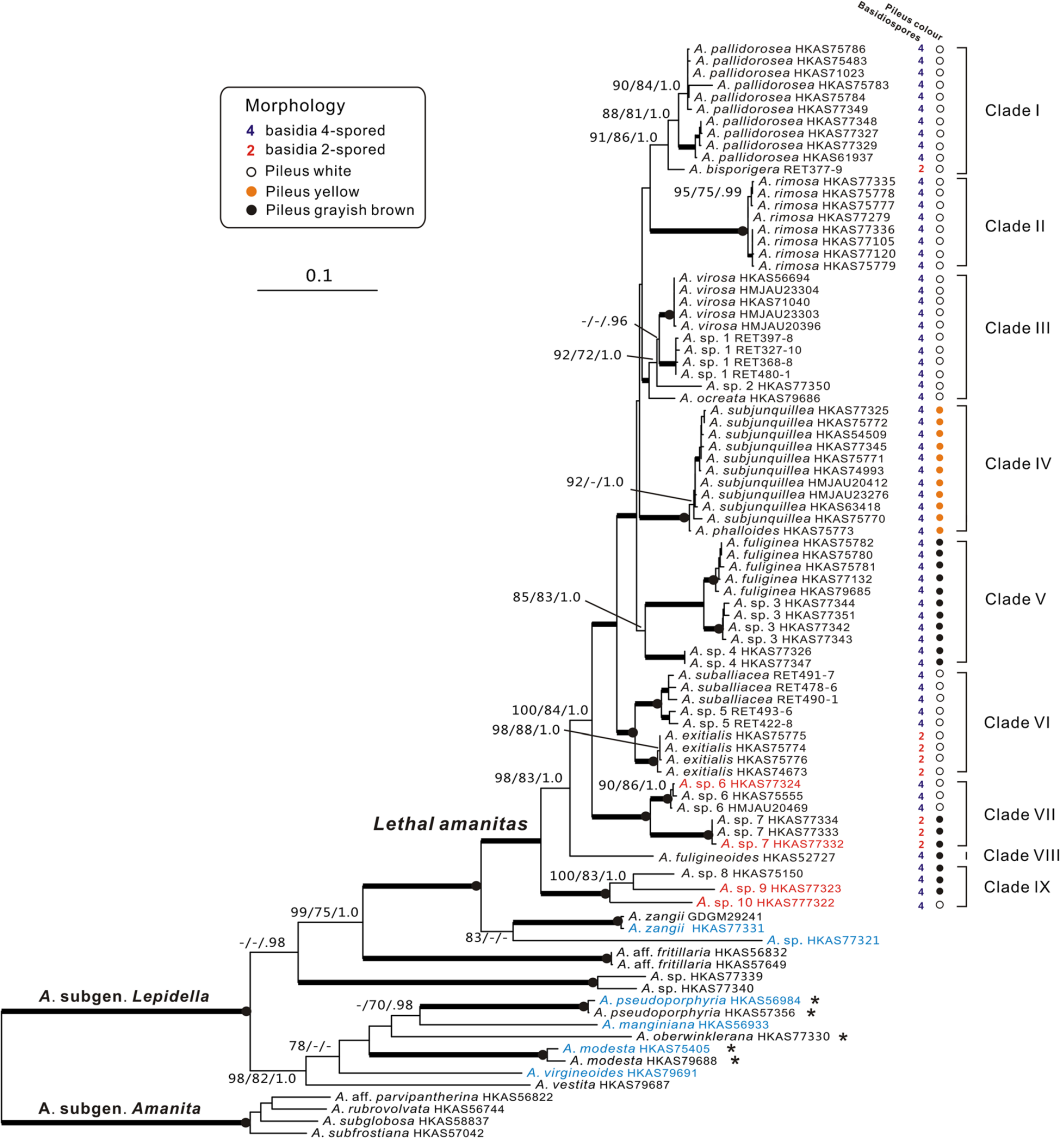
**Phylogenetic tree inferred from maximum likelihood (ML) analysis based on the combined dataset (nrLSU, *rpb2, ef1*-α and β-*tubulin*).** Only maximum likelihood bootstraps (LB) and maximum parsimony bootstraps (PB) over 70%, and Bayesian posterior probabilities (PP) over 0.90 are reported on the branches. Thickened branches indicate LB and PB between 90%–100%, and PP between 0.95–1.0. Thickened branches with dots on the root nodes represent 100% LB/PB and 1.0 PP values. The species names in red and blue colour indicate that toxins were detected and were not detected respectively in our biochemical analyses. Species, which were previously allocated in the section *Phalloideae* and later nested in the section *Lepidella*, are highlighted by asterisks after their names.

**Figure 2:**
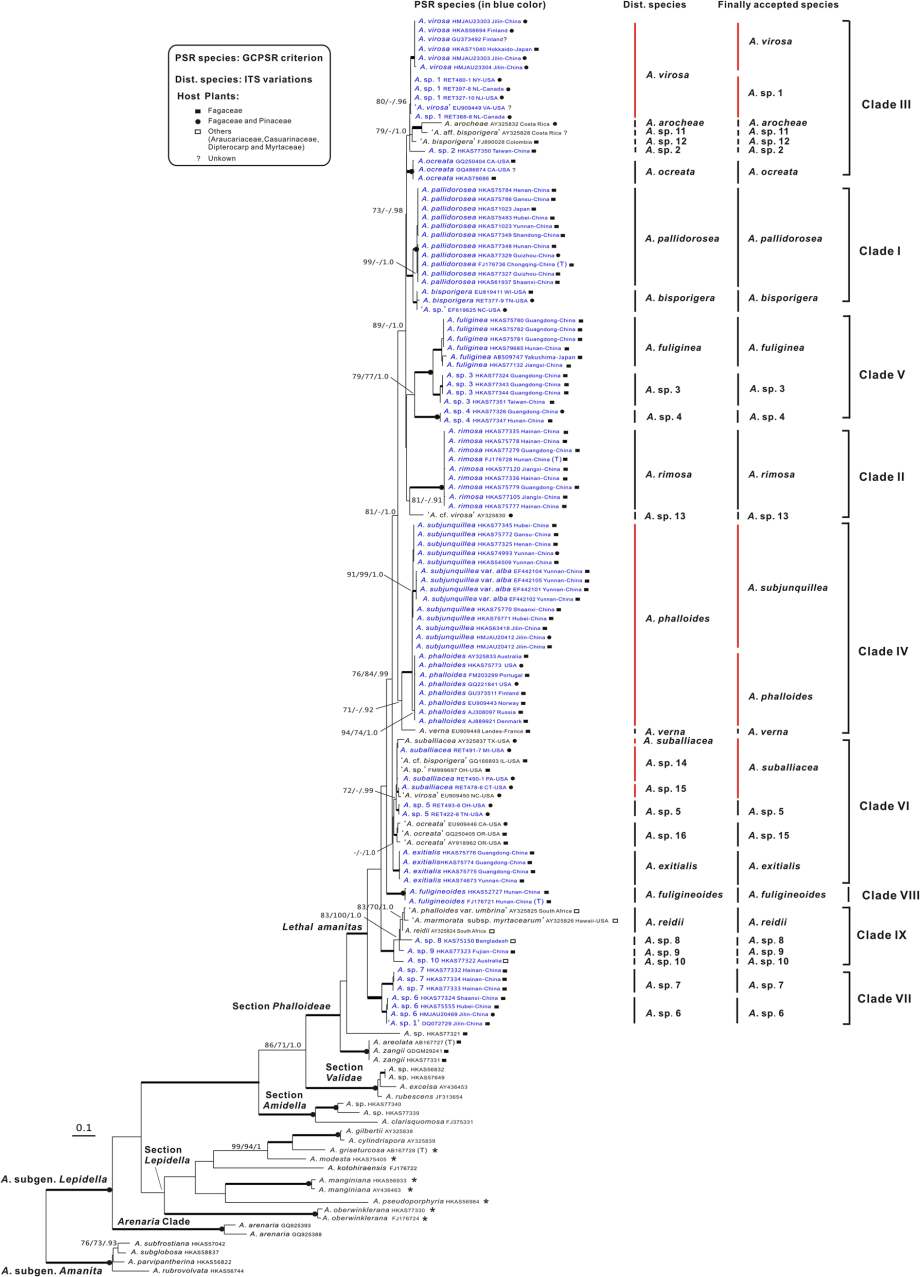
**Phylogenetic tree inferred from maximum likelihood (ML) analysis based on ITS sequences.** Only LB and PB over 70%, and PP over 0.90 were reported. Thickened branches indicate LB/PB between 90%–100% and PP between 0.95–1.0. Thickened branches with dots on the root nodes represent 100% PB/LB and 1.0 PP values. PSR species were identified according to the GCPSR criterion, the Dist. species were recognised by the variations of ITS sequences, and the Finally accepted species were defined by integrating molecular phylogenetic analyses with morphological studies. Species previously allocated in the section *Phalloideae* and later nested in the section *Lepidella* are highlighted by asterisks after their names. Sequences from type collections are indicated by (T).

The combined dataset (nrLSU, *rpb2*, *ef1*-α, and β-*tubulin*), in which the aligned lengths of the four gene loci were 896, 678, 573 and 428 bp respectively, contained 692 parsimony informative sites after excluding 341 bp constituting introns and ambiguously aligned sites. The tree obtained from the ML analysis with LB, PB and PP support based on the dataset is shown in Figure 1. The aligned ITS matrix comprised 850 positions with 502 parsimony-informative sites. The tree inferred from the ML analysis together with LB, PB and PP is shown in Figure 2. Considering only supported clades (PB > 70%, LB > 70% and PP > 95%), the backbone resolution of the phylogenetic tree generated from the combined dataset was higher than the tree inferred from ITS. Although the phylogenetic relationships among sections or species in the ITS tree were not well resolved, the good support in the terminal clades allowed us to delineate species boundaries. In that case, the ITS sequences could also be proposed as a DNA barcode marker for lethal amanitas.

In all of the analyses, putative lethal amanitas were clustered into two distinctive clades. The majority of them formed a monophyletic group included in the section *Phalloideae*, whereas *A. griseoturcosa* T. Oda et al., *A. manginiana sensus* W. F. Chiu, *A. modesta* Corner & Bas, *A. oberwinklerana* Zhu L. Yang & Yoshim. Doi and *A. pseudoporphyria* Hongo were nested in the section *Lepidella* with high statistical supports (Figures 1 and 2).

### Phylogenetic species recognition

Among all of the recognised phylogenetic lineages of lethal amanitas, 21 of them fulfilled the GCPSR criterion. Our rationale for provisionally recognising three of the remaining lineages, *A*. sp. 13 (‘*A*. cf. *virosa*’ AY325830), *A*. sp. 15 (‘*A. ocreata*’ EU909446, GQ250405, AY918962) and *A. verna* (Bull.: Fr.) Lam. (*A. verna* EU909448), was based on the fact that they were highly divergent from their sister groups. According to the second identification criterion based on the ITS sequences, the highest intraspecific variation of those reference species was 0.006 (Additional file 5: Table S4). Taking this value as a threshold, 28 species were recognised (Figure 2). Although *A. virosa* and *A*. sp. 1 were identified as different species according to the GCPSR criterion, they were defined as a single species according to the ITS variations because their interspecific divergence was equal to 0.006. In addition, the interspecific divergence between two lineages, *A. suballiacea* RET491-7 and *A. suballiacea* RET490-1/*A*. sp. FM999697/*A.* cf. *bisporigera* GQ166893, was also equal to 0.006, and thus they were combined as a single species. In contrast, the intraspecific divergence of provisionally adopted ‘*A. ocreata*’ EU909446/GQ250405/AY918962 and *A*. sp. 7 were respectively higher than the cutoff value, but they were both defined as a single species because the two species could not been split into more well supported subclades. Based on the two criteria in combination with morphological and anatomical analyses, 28 phylogenetic species were ultimately accepted.

### Biochemical analyses

As shown in Table 1 and Additional file 6: Figure S2, *A. exitialis*, *A. ocreata*, *A*. sp. 6, *A*. sp. 7, *A*. sp. 9 and *A*. sp. 10 exhibited the presence of amatoxins or phallotoxins. Yet, we failed to detect either amatoxins or phallotoxins in *A. flavipes* S. Imai, *A. manginiana*, *A. modesta*, *A. pseudoporphyria*, *A*. sp. HKAS77321, *A*. sp. HKAS79690, *A. virgineoides* Bas and *A. zangii* Zhu L. Yang et al.

**Table 1:** **Analysis of amatoxins and phallotoxins in representative *Amanita *** **species**

**Taxon**	**Section**	**Locality**	**Voucher**	**α-AMA**	**β-AMA**	**PHD**
*A. exitialis*	*Phalloideae*	Guangdong, China	HKAS38162	+	+	+
*A. flavipes*	*Validae*	Yunnan, China	HKAS79689	–	–	–
*A. manginiana*	*Lepidella*	Yunnan, China	HKAS56933	–	–	–
*A. modesta*	*Lepidella*	Guangdong, China	HKAS75405	–	–	–
*A. ocreata*	*Phalloideae*	California, USA	HKAS79686	+	+	+
*A. pseudoporphyria*	*Lepidella*	Yunnan, China	HKAS56984	–	–	–
*A.* sp.	*Phalloideae*	Yunnan, China	HKAS77321	–	–	–
*A.* sp. 6	*Phalloideae*	Shaanxi, China	HKAS77324	+	+	–
*A.* sp. 7	*Phalloideae*	Hainan, China	HKAS77332	–	+	–
*A.* sp*.* 9	*Phalloideae*	Fujian, China	HKAS77323	–	–	+
*A.* sp*.* 10	*Phalloideae*	Tasmania, Australia	HKAS77322	–	–	+
*A.* sp.	*Amidella*	Jiangxi, China	HKAS79690	–	–	–
*A. virgineoides*	*Lepidella*	Shandong, China	HKAS79691	–	–	–
*A. zangii*	*Phalloideae*	Fujian, China	HKAS77331	–	–	–

α-AMA = α-amanitin; β-AMA = β-amanitin; PHD = phalloidin.

– indicates no or extremely small quantities of toxins were detectable; + indicates that toxins were detected.

### Bayesian estimation of divergence times and historical biogeography of lethal amanitas

The alignment of the combined nrLSU, *rpb2* and *ef1*-α dataset, which was compiled only for the molecular dating analysis, consisted of 57 sequences 2428 bp in length. The aligned ITS dataset established to estimate the divergence time and biogeographical history of lethal amanitas was 739 bp in length with 201 parsimony informative sites.

Analyses calibrated by the first point, 582 Mya between Ascomycota and Basidiomycota, estimated the divergence time of *Amanita* at 158.47 ± 0.59 Mya (116.63–200.71 Mya, 95% HPD) that was close to the conservatively initial divergence time of the host plant family Pinaceae [70], and the initial diversification of lethal amanitas was at 64.84Mya (46.87–86.50Mya, 95% HPD) which was also at the similar time as the divergence of the host families Fagaceae, Casuarinaceae and Dipterocarpaceae [71-73]. Based on the second calibration point *Q. cranhamii*, the divergence time between Ascomycota and Basidiomycota was estimated to be at 285.66 ± 1.07 Mya (227.63–346.27 Mya, 95% HPD) which was much less than the minimal divergence age of Ascomycota/Basidiomycota (400 Mya). Meanwhile, the crown ages of *Amanita* and lethal amanitas estimated by the calibration point were around 79.77 ± 0.31 (64.39–96.60 Mya, 95% HPD) and 32.86 ± 0.18Mya (24.77–41.13 Mya, 95% HPD), respectively. The two values were also significantly lower than the estimated conservative divergence time of Pinaceae, Fagaceae, Casuarinaceae and Dipterocarpaceae. As a result, the second calibration point seemed to be vastly underestimate the divergence time of *Amanita* and lethal amanitas*.* Therefore, the first calibration point was eventually applied and the divergence time of lethal amanitas inferred from it is taken in the further analyses. The chronogram and estimated divergence time of *Amanita* from the calibration point is shown in Additional file 7: Figure S3.

**Figure 3:**
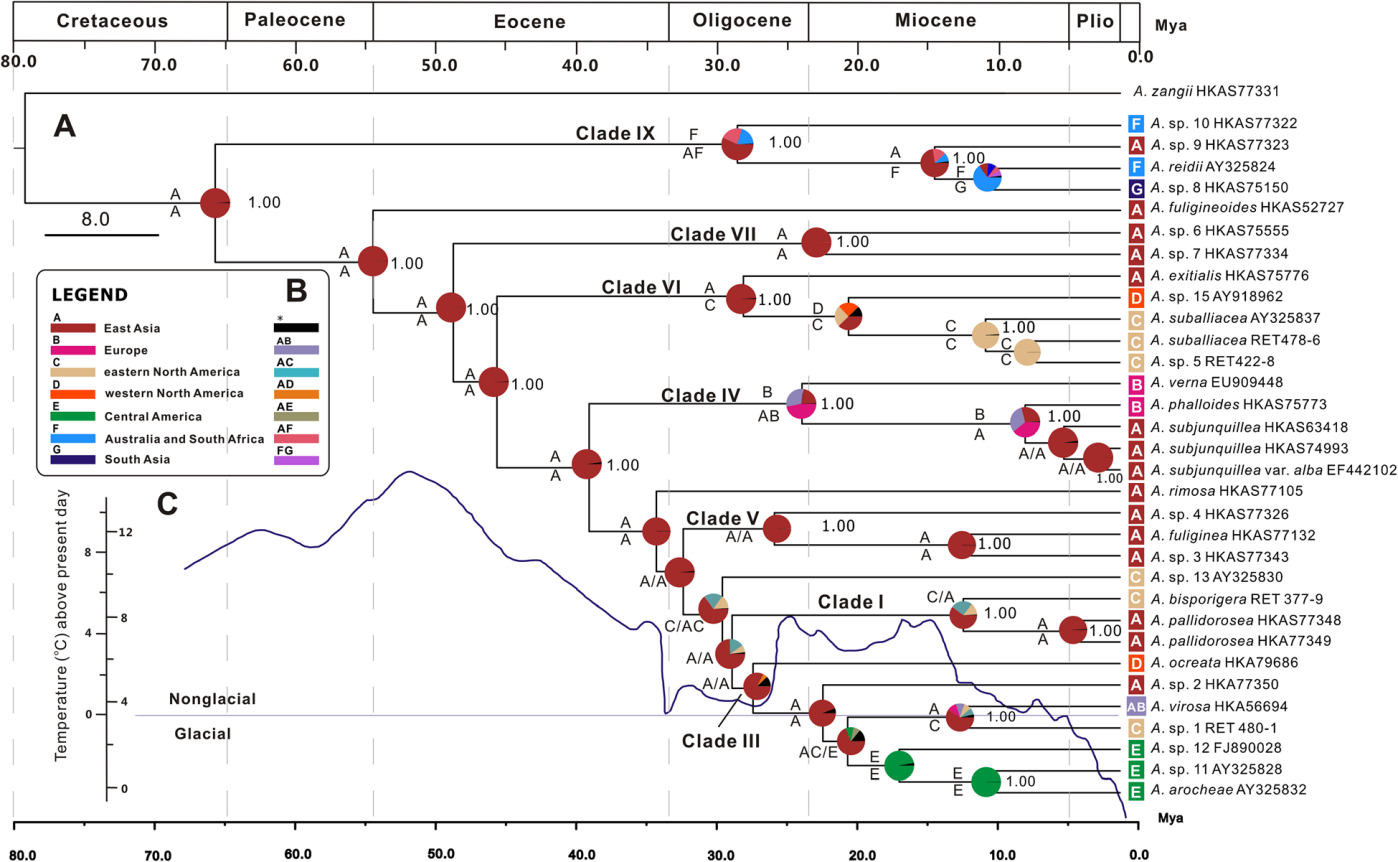
**Divergence time estimation and ancestral area reconstruction of lethal amanitas using the ITS dataset.** The chronogram was obtained from the molecular clock analysis using BEAST. **(A)** Pie chart in each node indicates the possible ancestral distributions inferred from a Bayesian Binary MCMC analysis implemented in RASP. The characters above and beneath each branch identify the possible ancestral distribution estimated by the maximum likelihood-based program LAGRANGE. Bayesian credibility values (PP) over 0.95 are indicated near the pie chart of the tree. **(B)** Colour key to possible ancestral range at different nodes; black with an asterisk represents other ancestral ranges. **(C)** Global temperature means in the geological history are shown by the curve adapted from Zachos et al. [74].

The inferred historical biogeographic scenarios from analyses using RASP and LAGRANGE are shown in Figure 3. Both the maximum likelihood-based estimation and Bayesian MCMC analyses strongly supported East Asia as the ancestral area of lethal amanitas. Our results also indicated four kinds of intercontinental distribution patterns of sister species or sister groups, viz. East Asia–eastern North America, Eurasia–western North America–eastern North America–Central America, East Asia–Europe, and southern East Asia–South Asia–Australia.

## Discussion

### *Recircumscription of* A*.* sect. Phalloideae

In our analyses, some unexpected relationships within the section *Phalloideae* were indicated. *Amanita griseoturcosa*, *A. manginiana*, *A. modesta*, *A. pseudoporphyria* and *A. oberwinklerana*, which were previously defined as lethal amanitas based exclusively on morphological studies, are phylogenetically nested in the section *Lepidella* (Figures 1 and 2), [3,8,9,22,75]. On the other hand, *A. areolata* T. Oda et al., *A. hesleri* Bas and *A. zangii* are robustly supported to form a monophyletic group with lethal amanitas (Figures 1 and 2), although they were originally allocated to the section *Lepidella* due to their felty to subfibrillose, adnate volval remnants on pilei, appendiculate pileal margins, and elongated stipes with indistinct bulb with farinose to floccose volval remnants [3,8,10,22]. Our biochemical analyses failed to detect either amatoxins or phallotoxins in *A. manginiana* (fresh or dried basidiomata of it has been sold as food in free markets in Yunnan for centuries [3]), *A. modesta*, *A. pseudoprphyria* (Table 1). We also did not detect any toxins in *A. zangii* and *A*. sp*.* HKAS77321 (Table 1). Consequently, the five species, *A. griseoturcosa*, *A. manginiana*, *A. modesta*, *A. pseudoporphyria* and *A. oberwinklerana*, which were previously treated as members of lethal amanitas, should be excluded from the section *Phalloideae* and then allocated to the section *Lepidella*. In contrast, *A. areolata*, *A. hesleri* and *A. zangii* should be transferred from the section *Lepidella* to the section *Phalloideae* as a basal lineage producing neither amatoxins nor phallotoxins. That kind of similar morphology among distantly related species may have resulted from the evolutionary convergence or from shared plesiomorphies, which is also suggested in other fungi [76,77]. Moreover, *A. zangii* and *A. areolata* are indistinguishable in our molecular phylogenetic analysis (Figure 2), and thus *A. areolata* should be regarded as a synonym of *A. zangii*, as suggested by Yang [3] based on morphological studies.

### Diversity of lethal amanitas

Nine major lineages comprising 28 phylogenetic species are supported within lethal amanitas (Figures 1 and 2). In the following discussion, we focus on the most significant features circumscribing the major clades and their distribution patterns.

**Figure 4:**
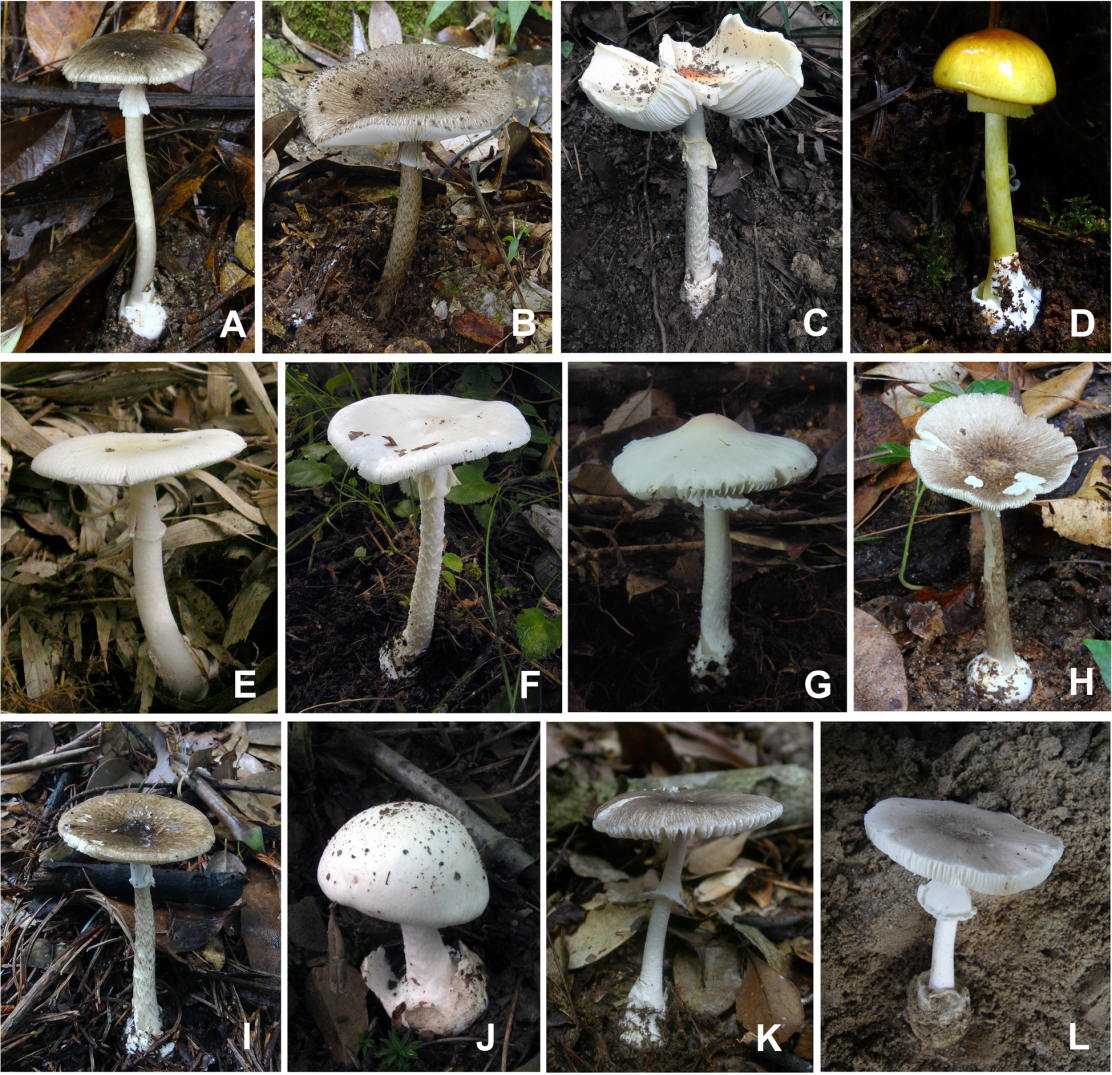
**Representatives of lethal amanitas. (A)***Amanita fuliginea*; **(B)***A. fuligineoides*; **(C)***A. pallidorosea*; **(D)***A. subjunquillea*; **(E)***A. rimosa*; **(F)***A. virosa*; **(G)***A.* sp. 2; **(H)***A.* sp. 3; **(I)***A.* sp. 4; **(J)***A.* sp. 6; **(K)***A.* sp. 7; and **(L)***A.* sp. 8.

Clade I includes two taxa, the East Asian species *A. pallidorosea* (C in Figure 4) and the eastern North American species *A. bisporigera* G. F. Atk. The two taxa share the characteristics of a white basidioma and globose to subglobose basidiospores. *Amanita pallidorosea* is widely distributed in East Asia from Yunnan, southwestern China to Jilin, northeastern China and Hokkaido, Japan under broad-leaved forests. Although *A. pallidorosea* is clustered into two subclades in all of the single-gene phylogenetic trees, it is ultimately delimited as a single species following a conservative approach, as the two subclades are not well supported in the phylogenetic trees inferred from both the nrLSU and ITS sequences (Figure 2 and Additional file 3: Figure S1), and the interspecific divergence between them is lower than the cut off values of ITS sequences variations. Zhang et al. [27] suggested that *A. pallidorosea*, excluding the white forms, could be distinguished from other East Asian white amanitas by its rose pileus with a conspicuous umbo over the disc. However, *A*. sp. 2 (G in Figure 4) in our study also possesses a pallid rose pileus with a noticeable umbo. Although *A. pallidorosea* and *A*. sp. 2 are morphologically similar to each other, they can be distinguished through microscopic analyses, because the spores of *A*. sp. 2, (7–) 7.5–10 (−11.5) × (7–) 7.5–9 (−10.5) μm, are larger than those of *A. pallidorosea* (6–) 6.5–8 (−10) × 6–7.5 (−9.5) μm. *Amanita bisporigera* is the sister species to *A. pallidorosea* in eastern North America. It is interesting to note that *A. pallidorosea* has four-spored basidia, while the basidia of *A. bisporigera* are two-spored.

Clade II consists of two species with a white basidioma, *A. rimosa* (E in Figure 4) from subtropical East Asia under Fagaceae trees, and *A*. sp. 13 (‘*A*. cf. *virosa*’ AY325830) from eastern North America. *Amanita rimosa* was initially suggested to be distinct from the other white lethal amanitas in its rimose pileal surface [27]. However, this feature is found to be variable and dependent on environmental conditions in our analyses. Compared with other currently known white lethal amanitas restricted to tropical and subtropical East Asia, *A. rimosa* has a much smaller and more slender basidioma, with a pileus of 1.5–5 cm in diameter.

Clade III includes seven phylogenetic species ranging from the Palearctic to the Nearctic. All of these species have a white basidioma with the exception of *A. arocheae* which has a grayish pileus. *Amanita virosa* (F in Figure 4) was originally described from northern Europe under mixed forests of Fagaceae and Pinaceae [5], and it has often been reported in eastern and southern Asia [27,78]. Based on our molecular and morphological analyses, *A. virosa* does occur in East Asia, but is restricted to its northeastern region. *Amanita virosa* was also reported from North America [21]. However, Tulloss et al. [79] questioned its distribution in North America. In our analyses, all of the North American collections closely related to *A. virosa* are clustered in their own lineage, *A*. sp. 1. Although the ITS sequences divergence between them is low, they are ultimately delimited as two different species because their monophyly was not well supported even in the combined analysis (Figures 1, 2 and Additional file 3: Figure S1). Therefore, *A. virosa* only occurs in the Palearctic regions (Europe and northeastern Asia) and its American counterpart might represent a new taxon. *Amanita ocreata* under the mixed forest of Fagaceae and Pinaceae is the only western North American species in this clade. Because *A*. sp. 2, *A*. sp. 11 and *A*. sp. 12 are all represented by a single sample, their definitions require further research.

Clade IV includes two European species, *A. verna* and *A. phalloides*, and one East Asian species, *A. subjunquillea* (D in Figure 4), with no close relatives known from North America to date. The three species are associated with mixed forest of Fagaceae, Pinaceae and Betulaceae throughout the broad-leaved forests of Europe and East Asia. *Amanita verna*, which has a white basidioma, is sister to *A. phalloides* and *A. subjunquillea* with moderate support values. *Amanita subjunquillea* and *A. phalloides* share the characteristic olive-green to yellow pileus. *Amanita phalloides* was originally described in Europe, and has been introduced to North America, Australia, New Zealand and South Africa together with its host plants [30,80]. It has also been reported in China [81] and Japan [75]. However, those collections identified as *A. phalloides* in China were found to be *A. subjunquillea*[3,27]. In East Asia, *A. subjunquillea* has often been mistaken as *A. phalloides* due to their high degree of morphological similarities and close phylogenetic relationship, but the spores, basidia and basidiomata of *A. subjunquillea* are usually smaller than those of *A. phalloides*[3,8].

Clade V consists of three sympatric species occurring with Fagaceae restricted to tropical and subtropical East Asia, *A. fuliginea*, *A*. sp. 3 and *A*. sp. 4 (A, H and I in Figure 4, respectively). Taxa of this clade are characterised by their small basidioma with a fuliginous to almost blackish pileus 2–6 cm in diameter. The similarity among them may result from the morphological stasis caused by stable and similar habitats, which has been proposed as a common phenomenon in the evolution of fungi [82-84]. Zhang et al. [27] suggested that there were two subclades of *A. fuliginea*, and interpreted one of the subclades (*A*. sp. 4 in Figures 1 and 2) as a different population or a cryptic species of *A. fuliginea*. However, in our analyses, the two subclades were suggested to be different species based on both the GCPSR criterion and the variation of ITS sequences (Figures 1 and 2). *Amanita fuligineoides* (B in Figure 4) in Clade VIII is also morphologically similar to *A. fuliginea*, but it has a larger-sized basidioma [27].

Clade VI includes four phylogenetic species characterised by white basidioma and subglobose, globose to ellipsoid basidiospores. The four species are largely associated with Fagaceae. In this clade, the delimitation of *A. suballiacea* is controversial. According to the ITS sequences, *A. suballiacea* AY325837, *A*. sp. 14 and *A*. sp. 16 are identified as different phylogenetic species. However, our morphological and microscopic analyses both indicate that the three collections of *A*. sp. 14 RET478-6, 490-1 and 491-7 are identical to *A. suballiacea*. Consequently, they are ultimately treated as a single species (Figure 2). Although the sequence divergence between *A.* sp. 5 and *A. suballiacea* is low, they have been proved to be different phylogenetic species by both the GCPSR and ITS sequences divergence criteria (Figures 1 and 2). *Amanita* sp. 15 (‘*A. ocreata*’ EU909446, GQ250405 and AY918962) from western North America was identified as *A. ocreata*, but the real *A. ocreata* is within Clade III in our analyses as proved by both morphological and anatomical evidence. Thus, *A*. sp. 15 represents an independent species. *Amanita exitialis*, which is restricted to tropical and subtropical East Asia, is distinct from its North American sister group in its two-spored basidia.

Clade VII contains two East Asian species, *A*. sp. 6 and *A*. sp. 7 (J and K in Figure 4), associated with mixed forests of Fagaceae and *Pinus. Amanita* sp. 6, with a white basidioma and four-spored basidia, was restricted to temperate East Asia. In contrast, *A*. sp. 7, with a grayish pileus and two-spored basidia, is only known from tropical East Asia.

Clade IX consists of four phylogenetic species occupying the basal position in the phylogenetic tree (Figure 1). Morphologically, the four species are characterised by their brown, grayish or grey-brown basidiomata with the exception of *A*. sp. 10, which has a white basidioma. *Amanita reidii* Eicker & Greuning was originally reported in South Africa under the introduced Australian plant *Eucalyptus* with a citation of the collection PREM 48618 from Sabie [85], which was erroneously recoded as ‘*Amanita phalloides* var. *umbrina*’ in GenBank [AY325825]. The Hawaiian *A. marmorata* subsp. *myrtacearum* O. K. Mill. et al. was observed in association with exotic trees of Myrtaceae, Casuarinaceae and Araucariaceae [7]. Hallen et al. [29] suggested that *A. reidii* might be synonymous with the Australian species *A. marmorata* subsp. *marmorata* Cleland & E.-J. Gilbert and the Hawaiian subspecies *myrtacearum*. Miller et al. [7] suggested that the three taxa, *A. marmorata* subsp. *marmorata*, *A. marmorata* subsp *myrtacearum* and *A. reidii*, were a complex of closely related taxa that might have originated in eastern Australia and been imported into the Hawaiian islands and South Africa with their host plants, with which we fully agreed. In our analyses, the three collections from Hawaii and South Africa showed intraspecific divergence lower than the cutoff value, and thus they were identified as a single species, *A. reidii* (Figure 2). *Amanita* sp. 10 was collected in Tasmania, Australia under *Casuarina*. Another two phylogenetic species, *A*. sp. 8 (L in Figure 4) and *A*. sp. 9, were collected in South Asia and tropical East Asia associated with Dipterocarpaceae and Fagaceae, respectively. The two species or their affinities are likely to be found in other parts of tropical Asia in future studies.

Although the relationships among the deeper clades of lethal amanitas are not well resolved, a few interesting features seem to have evolved convergently (Figures 1 and 2). For example, species with two-spored basidia such as *A. exitialis*, *A. bisporigera* and *A*. sp. 7 do not have close relationships to each other. Instead, they are closely related to taxa with four-spored basidia. Furthermore, species with unpigmented and pigmented pilei do not correspondingly form monophyletic groups but some of them are clustered together, such as *A*. sp. 7 (with a gray-brown pileus) and *A*. sp. 6 (with a white pileus) (Clade VII in Figures 1 and 2). It could be speculated that the characteristics of two-spored basidia and unpigmented/pigmented pilei have evolved independently several times within lethal amanitas. Yet, the presence of lethal substances within *Amanita* is suggested to have a single origin and it seems to be a synapomorphy of lethal amanitas, because our biochemical analyses show that the sister species of lethal amanitas and those samples from the other three sections of the subgenus *Lepidella* contained no detectable cyclic peptide toxins (Table 1). That is also consistent with the studies of Hallen et al. [86], in which they proposed that the lack of toxin production among other species of *Amanita* outside of section *Phalloideae* were due to the absence of encoding genes.

### Divergence time within lethal amanitas and their intercontinental distribution patterns

Our analyses show that the divergence times estimated by the two fossils, which should be consistent, are greatly different. The second calibration point (*Q. cranhamii*) seems to have vastly underestimated the divergence time of Ascomycota/Basidiomycota and *Amanita*. That might be resulted from *Q. cranhamii* representing a relatively young taxon of *Quatsinoporites*, or that the hypothesised position of this fossil taxon within Basidiomycota requires further verification, as the phylogenetic position of the fossil appears to have great influence over the estimation results. For example, the estimated divergence time between Ascomycota and Basidiomycota varied from 452 Mya to 582 Mya with the calibration point *P. devonicus* placed in different subphyla of Ascomycota [62,87].

In our biogeographical analyses, those lethal amanitas in the basal and sub-basal groups were collected in tropical East Asia, South Asia, South Africa and Australia, showing a palaeotropical distribution pattern. Furthermore, the sister species of lethal amanitas, *A. zangii* and *A*. sp. HKAS77321, were also collected in the palaeotropical areas (tropical East Asia). These findings strongly suggest a possible palaeotropical origin of lethal amanitas, which has also been suggested for other ECF such as Hysterangiales [88], Inocybaceae [89] and Porcini mushrooms [90]. In the basal group, *A*. sp. 10 and *A. reidii* were collected in Australia, South Africa and Hawaii associated with Araucariaceae, Casuarinaceae and Mytraceae, and *A*. sp. 8 was collected in Bangladesh under *Shorea robusta*[7,85]. In addition, there are also about seven lethal amanitas reported in Madagascar, the Congo and South America associated with Fabaceae and *Nothofagus*[25,91,92]. Unfortunately, we know of no collections of these species except types which are not suitable for molecular phylogenetic studies. However, according to the coevolution of fungi and host plants, the Gondwana origin can not be rejected because Araucariaceae, Dipterocarpaceae, *Nothofagus* and Myrtaceae were all suggested to have a Gondwana origin [93-97].

Three independent sister species or sister groups among Eurasia/East Asia and the Americas are indicated in the ancestral area reconstructions analyses (Figures 1, 2 and 3). The first species pair is within Clade I, which exhibits an East Asian–eastern North American disjunct distribution (Graysian distribution, [2]) (Figures 1 and 3), and the estimated divergence time between them is about 11.4 Mya (1.11–13.84 Mya, 95% HPD), in the late Miocene. The second intercontinental distribution among Eurasia and North/Central America is exhibited in Clade III. The dated divergence time of the western American species *A. ocreata*, which occupies the basal position in the clade, is about 26 Mya (17.74–35.72 Mya, 95% HPD) in the late Oligocene. In addition, the divergence between the Eurasian species *A. virosa* and its eastern North America counterpart (*A*. sp. 1) is estimated to occur about 13 Mya (3.75–19.84 Mya, 95% HPD) in the middle Miocene. The other three Central American species in the clade, *A. arocheae*, *A*. sp. 11 and *A*. sp. 12, diversified during 15.7–10 Mya. Due to their close relationships with the North American species and their relatively recent divergence, we speculate that they originated in the northern part of the Americas and then extended into Central America with their host plants oaks, which had a North Temperate origin [2,98]. The third species group which exhibited the East Asia and North/Central America disjunct distribution pattern is within clade VI, with one East Asian, one western North American and two eastern North American species. The estimated divergence time between the East Asian taxon *A. exitialis* and its American sister group is around 27 Mya (8.88–46.58 Mya) in the middle Oligocene, and the divergence time between the western North America species *A*. sp. 15 and its eastern North America sister group is around 19.36 Mya in the early Miocene.

Our results suggest that the intercontinental distribution patterns of the sister species or sister groups among Eurasia/East Asia and the Americas were mainly established during the middle Oligocene to the middle Miocene, which coincides with the paleoclimates. Since the climatic deterioration at 33 Mya, the temperature began to fluctuate from the early Oligocene to the middle Miocene (34–15 Mya) [99]. The fluctuation of temperature, especially the relatively warm climates from the late Oligocene to the middle Miocene, may have allowed temperate elements to migrate between continents via the Bering Land Bridge (BLB). This biogeographic distribution pattern was elucidated in *A. muscaria*[100], and was also consistent with the diversification of the major host plant family in the Northern Hemisphere, Fagaceae, which appeared to have achieved a continuous distribution spanning Asia, North America, and Europe during the Oligocene through the floristic exchanges via the North Atlantic Land Bridge before the lower Oligocene and later via the BLB, followed by allopatric speciation in the middle Miocene due to the climate change [71].

In Clade IX, the four lethal amanitas from southern East Asia, Hawaii, Australia and South Africa exhibit close relationships. *Amanita reidii* might have originated from Australia and then been introduced to South Africa with the host plants *Eucalyptus*[7,85]. The sister group relationships among the tropical East Asian species *A*. sp. 9, South Asian species *A*. sp. 8 and *A. reidii* are not surprising, as the two continents were connected after the collision of the Australian and Asian plates in the Miocene [101,102]. However, the estimated divergence time of *A*. sp. 10 and the other species in the clade, 27 Mya, predates the Miocene collision between Australia and Asia. *Amantia* sp. 10 might be an old relic of lethal amanitas in the palaeotropical regions because it showed great divergence from other lethal amanitas and occupied a basal position in the phylogenetic tree (Figures 1 and 3). The same may be true for *A. rimosa* and *A. fuligineoides*, which showed significant divergence from other lethal amanitas and occupied an isolated position in the phylogenetic tree (Figures 1 and 3).

Our results also confirm an East Asian–European allopatric speciation, viz. *A. subjunquillea* and *A. phalloides*. The sequence variations between them are relatively low even in the multi-locus analysis, which indicates a recent divergence, 7 Mya (2.04–12.91 Mya, 95% HPD). It is probable that the divergence of *A. subjunquillea* and *A. phalloides* was brought about by the vicariance of a recent common ancestral distribution in the Holarctic region. Later, in the late Miocene, the common ancestor moved southward with their host plants because of the distinct climatic cooling in that period [99,103], and then diverged into two regional species. These findings indicate that the dispersal-vicariance theory, which has been widely used to explain the disjunctions of plants between the Palearctic and Nearctic regions [104], is applicable in understanding the intercontinental distribution patterns of ECF.

In our molecular phylogenetic analyses, the relationships among the temperate-subtropical clades (Clades I–V, Figure 1) were not well resolved. That could also be explained by the paleoclimatic changes. The gradual global cooling after 50 Mya in the Eocene may have stimulated the early diversification of lethal amanitas into their major extant tropical to temperate clades after origination in the early Paleocene in Palaeotropical areas, whereas the climate deterioration at 33 Mya may have led to an elimination of tropical elements (Figure 3) [103,105]. That the molecular phylogenetic analyses could not resolve the relationships among those temperate-subtropical clades may be explained by the extinction of tropical species obscuring the relationships among temperate-subtropical clades, or by the rapid speciation of temperate-subtropical species triggered by ecological changes.

## Conclusion

For the first time, a comprehensive phylogenetic study of lethal amanitas has been constructed using sequences of five gene fragments with an emphasis on samples from the Northern Hemisphere. Our results strongly supported the monophyly of lethal amanitas and indicated a single origin of the cyclic peptide toxins within *Amanita*. Twenty eight phylogenetic species of lethal amanitas were revealed, and half of them were proved to be potentially new. Our ancestral area reconstructions and analyses suggested that lethal amanitas probably originated in the Palaeotropical zone at about 64.92 Mya in the Paleocene. The intercontinental distribution patterns of sister species or sister groups among Eurasia and Americas, or among East Asia and the Americas were probably established during the middle Oligocene to Miocene via the BLB, followed by allopatric speciation caused by vicariance. Furthermore, our study highlighted the need for more molecular-phylogenetic studies on collections from the tropics and the Southern Hemisphere.

## Availability of supporting data

The data sets supporting the results of this article are available in the Dryad Digital Repository (doi:10.5061/dryad.8db34) [106].

## Competing interests

The authors declare that they have no competing interests.

## Authors’ contributions

ZLY and QC conceived and designed the experiments; QC, RET, LPT, BT PZ and ZLY provided the materials; QC generated the DNA sequences and analysed the data; QC and ZHC carried out the cyclic peptide toxins analyses. ZLY and RET carried out the taxonomic studies. QC, RET, LPT, and ZLY wrote the paper. All of the authors approved the final submission. All authors read and approved the final manuscript.

## Supplementary Material

Additional file 1: Table S1 Voucher information and GenBank accession numbers of the samples used in this study. Click here for file

Additional file 2: Table S2 GenBank accession numbers of the downloaded sequences used in the phylogenetic analyses. Click here for file

Additional file 3: Figure S1 Phylogenetic trees inferred from the maximum likelihood (ML) analysis with branch support obtained by ML and BI analyses based on nrLSU, *rpb2*, *ef1*-α and β-*tubulin* sequences, respectively. Click here for file

Additional file 4: Table S3 GenBank accession numbers of the sequences used in the divergence time estimation. Click here for file

Additional file 5: Table S4 The average evolutionary divergence over ITS sequence pairs within and between groups (provisionally adopted phylogenetic species) calculated by MEGA 4 using the Maximum Composite Likelihood model. Click here for file

Additional file 6: Figure S2 Results of the HPLC analyses. Click here for file

Additional file 7: Figure S3 Chronogram and estimated divergence times of lethal amanitas generated from the molecular clock analysis using the combined (nrLSU/*rpb2*/ *ef1*-α) dataset. Click here for file
