# Supplementary material for: Multi-locus phylogeny of lethal amanitas: Implications for species diversity and historical biogeography
Source: BMC Evol Biol. 2014 Jun 21;14:143. doi: 10.1186/1471-2148-14-143 (PMC4094918; doi:10.1186/1471-2148-14-143)
Supplement: Additional file 1: Table S1 — Voucher information and GenBank accession numbers of the samples used in this study. [file 1471-2148-14-143-S1.pdf]

Table S1 Voucher information and GenBank accession numbers of samples used in this study.

Taxa	Voucher	Locality	nrLSU	ITS	*rpb2*	*ef1-α*	*β-tubulin*
**Ingroup**							
A. bisporigera	RET 377-9 (duplicate HKAS77811)	Great Smoky Mountains National Park, Tennessee, USA	KJ466434	KJ466374	---	KJ481936	KJ466501
A. exitialis	HKAS74673	Kunming, Yunnan, China	KJ466435	KJ466375	KJ466590	KJ481937	KJ466502
A. exitialis	HKAS75774	Baiyunshan, Guangdong, China	JX998052	JX998027	KJ466591	JX998001	KJ466503
A. exitialis	HKAS75775	Fenghuangshan, Guangdong, China	JX998053	JX998026	KJ466592	JX998002	KJ466504
A. exitialis	HKAS75776	Guangzhou, Guangdong, China	JX998051	JX998025	KJ466593	JX998003	KJ466505
A. fuliginea	HKAS75780	Fengkai, Guangdong, China	JX998048	JX998023	KJ466595	JX997995	KJ466507
A. fuliginea	HKAS75781	Fengkai, Guangdong, China	JX998050	JX998021	KJ466596	JX997994	KJ466508
A. fuliginea	HKAS75782	Fengkai, Guangdong, China	JX998049	JX998022	KJ466597	JX997996	KJ466509
A. fuliginea	HKAS77132	Fuzhou, Jiangxi, China	KJ466436	KJ466375	KJ466598	KJ481939	KJ466510
A. fuliginea	HKAS79685	Hunan, China	KJ466437	KJ466376	KJ466594	KJ481938	KJ466506
A. fuligineoides	HKAS52727	Hunan, China	JX998047	JX998024	KJ466599	---	KJ466511
A. ocreata	HKAS79686	Contra Costa County, California, USA	KJ466442	KJ466381	KJ466607	KJ481947	KJ466518
A. pallidorosea	HKAS77327	Daoxian, Guizhou, China	KJ466446	KJ466386	KJ466608	KJ481948	KJ466519
A. pallidorosea	HKAS61937	Taibai Mountain, Shaanxi, China	KJ466443	KJ466382	KJ466609	KJ481949	KJ466520
A. pallidorosea	HKAS71023	Hokkaido, Japan	KJ466444	KJ466383	KJ466624	KJ481960	KJ466536
A. pallidorosea	HKAS75483	Shennongjia, Hubei, China	KJ466445	KJ466384	KJ466623	KJ481959	KJ466535
A. pallidorosea	HKAS75783	Nujiang, Yunnan, China	JX998055	JX998035	KJ466625	JX998010	KJ466537
A. pallidorosea	HKAS75784	Neixiang, Hennan, China	JX998056	JX998036	KJ466626	JX998009	KJ466538
A. pallidorosea	HKAS75786	Tianshui, Gansu, China	JX998054	JX998037	KJ466627	JX998011	KJ466539
A. pallidorosea	HKAS77329	Daoxian, Guizhou, China	KJ466447	KJ466387	KJ466610	KJ481950	KJ466521
A. pallidorosea	HKAS77348	Hunan, China	KJ466448	KJ466387	KJ466611	KJ481951	KJ466522
A. pallidorosea	HKAS77349	Mount Tai, Shandong, China	KJ466449	KJ466389	KJ466628	KJ481961	KJ466540
A. phalloides	HKAS75773	California, USA	JX998060	JX998031	KJ466612	JX998000	KJ466523

A. rimosa	HKAS75777	Baisha, Hainan, China	JX998044	JX998018	KJ466615	JX998005	KJ466526
A. rimosa	HKAS75778	Qiongzhong, Hainan, China	JX998045	JX998019	KJ466616	JX998006	KJ466527
A. rimosa	HKAS75779	Chebaling, Gunagdong, China	JX998046	JX998020	KJ466617	JX998004	KJ466528
A. rimosa	HKAS77105	Fuzhou, Jiangxi, China	KJ466452	KJ466391	KJ466618	KJ481954	KJ466529
A. rimosa	HKAS77120	Fuzhou, Jiangxi, China	KJ466453	KF479044	KJ466619	KJ481955	KJ466530
A. rimosa	HKAS77279	Fengkai, Guangdong, China	KJ466454	KJ466392	KJ466620	KJ481956	KJ466531
A. rimosa	HKAS77335	Qiongzhong, Hainan, China	KJ466455	KJ466393	KJ466621	KJ481957	KJ466532
A. rimosa	HKAS77336	Zhaoqing, Guangdong, China	KJ466456	KJ466394	KJ466622	KJ481958	KJ466533
A. sp. 1	RET 327-10 (duplicate HKAS77812)	Newfoundland and Labrador, Canada	KJ466458	KJ466396	KJ466629	KJ481962	KJ466541
A. sp. 1	RET 368-8 (duplicate HKAS77813)	Newfoundland and Labrador, Canada	KJ466459	KJ466397	---	KJ481963	KJ466542
A. sp. 1	RET 397-8 (duplicate HKAS77814)	Belleplain State Forest, New Jersey, USA	KJ466460	KJ466398	---	KJ481964	KJ466543
A. sp. 1	RET 480-1 (duplicate HKAS77817)	Franklin County, New York, USA	KJ466461	KJ466399	KJ466630	KJ481965	KJ466544
A. sp. 2	HKAS77350	Taiwan, China	KJ466462	KJ466400	KJ466631	KJ481966	KJ466545
A. sp. 3	HKAS77342	Fengkai, Guangdong, China	KJ466463	KF479045	KJ466632	KJ481967	KJ466546
A. sp. 3	HKAS77343	Fengkai, Guangdong, China	KJ466464	KJ466401	KJ466633	KJ481968	KJ466547
A. sp. 3	HKAS77344	Fengkai, Guangdong, China	KJ466465	KJ466402	KJ466634	KJ481969	KJ466548
A. sp. 3	HKAS77351	Taiwan, China	KJ466466	KJ466403	KJ466635	KJ481970	KJ466549
A. sp. 4	HKAS77326	Fengkai, Guangdong, China	KJ466467	KJ466404	KJ466636	KJ481971	KJ466550
A. sp. 4	HKAS77347	Lechang, Guangdong, China	KJ466468	KJ466405	KJ466637	KJ481972	KJ466551
A. sp. 5	RET 422-8 (duplicate HKAS77816)	Somerset County, New Jersey, USA	KJ466469	KJ466406	KJ466649	KJ481983	KJ466563
A. sp. 5	RET 493-6 (duplicate HKAS77822)	Columbiana County, Ohio, USA	KJ466470	KJ466407	KJ466650	KJ481984	KJ466564
A. sp. 6	HKAS75555	Shennongjia, Hubei, China	KJ466471	KJ466408	KJ466638	KJ481973	KJ466552

A. sp. 6	HKAS77324	Taibai Mountain, Shaanxi, China	KJ466472	KJ466409	KJ466639	KJ481974	KJ466553
A. sp. 6	HMJAU20469	Changbai Mountain, Jilin, China	KJ466473	KJ466410	KJ466640	KJ481975	KJ466554
A. sp. 7	HKAS77332	Qiongzhong, Hainan, China	KJ466474	KJ466411	---	KJ481992	KJ466578
A. sp. 7	HKAS77333	Baisha, Hainan, China	KJ466475	KJ466412	KJ466660	KJ481993	KJ466579
A. sp. 7	HKAS77334	Lingshui, Hainan, China	KJ466476	KJ466413	KJ466661	KJ481994	KJ466580
A. sp. 8	HKAS75150	Gazipur, Bangladesh	KJ466477	KJ466414	KJ466641	KJ481976	KJ466555
A. sp. 9	HKAS77323	Fuzhou, Jiangxi, China	KJ466478	KJ466415	KJ466642	KJ481977	KJ466556
A. sp. 10	HKAS77322	Tasmania, Australia	KJ466457	KJ466395	KJ466643	KJ481978	KJ466557
A. suballiacea	RET 478-6 (duplicate HKAS77819)	Clarion County, Pennsylvania, USA	KJ466484	KJ466419	KJ466600	KJ481940	KJ466512
A. suballiacea	RET 490-1 (duplicate HKAS77820)	Middlesex County, Connecticut, USA	KJ466485	KJ466420	KJ466601	KJ481941	KJ466513
A. suballiacea	RET 491-7 (duplicate HKAS77821)	Macomb County, Michigan, USA	KJ466486	KJ466421	KJ466602	KJ481942	KJ466514
A. subjunquillea	HKAS54509	Kunming, Yunnan, China	KJ466487	KJ466422	---	KJ481985	KJ466568
A. subjunquillea	HKAS63418	Changbai Mountain, Jilin, China	KJ466488	KJ466423	KJ466651	KJ481986	KJ466569
A. subjunquillea	HKAS74993	Nujiang, Yunnan, China	KJ466489	KJ466424	KJ466652	KJ481987	KJ466570
A. subjunquillea	HKAS75770	Taibai Mountain, Shaanxi, China	JX998062	JX998034	KJ466653	JX997999	KJ466571
A. subjunquillea	HKAS75771	Shennongjia, Hubei, China	JX998063	JX998032	KJ466654	JX997997	KJ466572
A. subjunquillea	HKAS75772	Tianshui, Gansu, China	JX998061	JX998033	KJ466655	JX997998	KJ466573
A. subjunquillea	HKAS77325	Neixiang, Henan, China	KJ466490	KJ466425	KJ466656	KJ481988	KJ466574
A. subjunquillea	HKAS77345	Shiyan, Hubei, China	KJ466491	KJ466426	KJ466657	KJ481989	KJ466575
A. subjunquillea	HMJAU20412	Changbai Mountian, Jilin, China	KJ466492	KJ466427	KJ466658	KJ481990	KJ466576
A. subjunquillea	HMJAU23276	Helong, Jilin, China	KJ466493	KJ466428	KJ466659	KJ481991	KJ466577
A. virosa	HKAS56694	Juva, Filand	JX998058	JX998030	KJ466664	JX998007	KJ466583
A. virosa	HKAS71040	Hokkaido, Japan	KJ466496	KJ466429	KJ466665	KJ481997	KJ466584
A. virosa	HMJAU20396	Changbai Mountain, Jilin, China	JX998059	JX998029	---	JX998008	KJ466585
A. virosa	HMJAU23303	Fusong, Jilin, China	KJ466497	KJ466430	KJ466666	KJ481998	KJ466586

A. virosa	HMJAU23304	Fusong, Jilin, China	KJ466498	KJ466431	KJ466667	KJ481999	KJ466587
**Outgroup**							
A. aff. *fritillaria*	HKAS56832	Baoshan, Yunnan, China	KJ466479	KJ466372	KJ466644	KJ481979	KJ466558
A. aff. *fritillaria*	HKAS57649	Dali, Yunnan, China	KJ466480	KJ466373	KJ466645	KJ481980	KJ466559
A. manginiana	HKAS56933	Dali, Yunnan, China	KJ466438	KJ466378	KJ466603	KJ481943	KJ466515
A. modesta	HKAS75405	Fengkia, Guangdong, China	KJ466439	KJ466379	KJ466604	KJ481945	KJ466517
A. modesta	HKAS79688	Fengkia, Guangdong, China	KJ466440	---	KJ466605	KJ481944	KJ466516
A. oberwinklerana	HKAS77330	Hainan, China	KJ466441	KJ466380	KJ466606	KJ481946	
A. aff. *parvipantherina*	HKAS56822	Baoshan, Yunnan, China	JN941163	JN943170	JQ031115	KJ482005	KJ466566
A. pseudoporphyria	HKAS56984	Dali, Yunnan, China	KJ466450	KC429050	KJ466614	KJ481953	KJ466525
A. pseudoporphyria	HKAS57356	Baoshan, Yunnan, China	KJ466451	---	KJ466613	KJ481952	KJ466524
A. rubrovolvata	HKAS56744	Yingjiang, Yunnan, China	JN943181	JN943181	JQ031117	KJ482002	KJ466534
A. sp.	HKAS77321	Kunming, Yunnan, China	KJ466481	KJ466416	KJ466646	---	KJ466560
A. sp.	HKAS77339	Inchon, South Korea	KJ466482	KJ466417	KJ466647	KJ481981	KJ466561
A. sp.	HKAS77340	Jiujiang, Jiangxi, China	KJ466483	KJ466418	KJ466648	KJ481982	KJ466562
A. subfrostiana	HKAS57042	Dali, Yunnan, China	JN941162	JN943173	JQ031118	KJ482003	KJ466565
A. subglobosa	HKAS58837	Lijiang, Yunnan, China	JN941152	JN943177	JQ031121	KJ482004	KJ466567
A. vestita	HKAS79687	Hainan, China	KJ466494	---	KJ466662	KJ481995	KJ466581
A. virgineoides	HKAS79691	Qingdao, Shandong, China	KJ466495	---	KJ466663	KJ481996	KJ466582
A. zangii	GDGM29241	Fujian, China	KJ466499	KJ466432	KJ466668	KJ482000	KJ466588
A. zangii	HKAS77331	Hainan, China	KJ466500	KJ466433	KJ466669	KJ482001	KJ466589
