# Supplementary material for: Multi-locus phylogeny of lethal amanitas: Implications for species diversity and historical biogeography
Source: BMC Evol Biol. 2014 Jun 21;14:143. doi: 10.1186/1471-2148-14-143 (PMC4094918; doi:10.1186/1471-2148-14-143)
Supplement: Additional file 2: Table S2 — GenBank accession numbers of the downloaded sequences used in the phylogenetic analyses. [file 1471-2148-14-143-S2.pdf]

Table S2 GenBank accession numbers of the downloaded sequences used in the phylogenetic analyses. Quotation marks are added to indicate the uncertain taxonomic positions.

Taxon	ITS	Locality	Source
**Ingroup**			
A. arocheae	AY325832	Costa Rica	Direct submission
'*A.* aff. *bisporigera*'	AY325828	Costa Rica	Direct submission
A. bisporigera	EF619625	Orange County, North Carolina, USA	Parrent et al. 2007
A. bisporigera	EU819411	Wisconsin, USA	Palmer et al. 2008
'*A. bisporigera*'	FJ890028	Colombia	Vagas et al. 2011
'*A.* cf. *bisporigera*'	GQ166893	Cook County, Illinois, USA	Direct submission
A. fuliginea	AB509747	Yakushima, Japan	Direct submission
A. fuligineoides	FJ176721	Hunan, China	Zhang et al. 2010
'*A. marmorata* subsp. *myrtacearum*'	AY325826	Hawaii, USA	Direct submission
'*A. ocreata*'	AY918962	Oregon, USA	Direct submission
'*A. ocreata*'	EU909446	Santa Rosa Island, California, USA	Pringle et al. 2009
A. ocreata	GQ250404	Sonoma County, California, USA	Direct submission
'*A. ocreata*'	GQ250405	Oregon, USA	Direct submission
A. ocreata	GQ486874	Santa Cruz Island, California, USA	Wolfe et al. 2010
A. pallidorosea	FJ176736	Chongqing, China	Zhang et al. 2010
A. phalloides	AY325833	Australia	Direct submission
A. phalloides	AJ308097	Voronej region, Russia	Direct submission
A. phalloides	AJ889921	Denmark	Direct submission
A. phalloides	EU909443	Kragero, Norway	Pringle et al. 2009
A. phalloides	FM203299	Portugal	Direct submission
A. phalloides	GQ221841	New Jersey, USA	Wolfe et al. 2010
A. phalloides	GU373511	Finland	Direct submission
'*A. phalloides* var. *umbrina*'	AY325825	South Africa	Direct submission

A. reidii	AY325824	South Africa	Direct submission
A. rimosa	FJ176728	Hunan, China	Zhang et al. 2010
‘*A.* sp.’	FM999697	Ohio, USA	Burke et al. 2009
‘*A.* sp. 1’	DQ072729	Jilin, China	Zhang et al. 2010
A. suballiacea	AY325837	Texas, USA	Direct submission
A. subjunquillea var. *alba*	EF442101	Yunnan, China	Li et al. 2007
A. subjunquillea var. *alba*	EF442102	Yunnan, China	Li et al. 2007
A. subjunquillea var. *alba*	EF442105	Yunnan, China	Li et al. 2007
A. subjunquillea var. *alba*	EF442104	Yunnan, China	Li et al. 2007
A. verna	EU909448	France	Direct submission
‘*A.* cf. *virosa*’	AY325830	New Jersey, USA	Direct submission
‘*A. virosa*’	EU909449	Virginia, USA	Pringle et al. 2009
‘*A. virosa*’	EU909450	North Carolina, USA	Pringle et al. 2009
A. virosa	GU373492	Finland	Direct submission
**Outgroup**			
A. areolata	AB167727	Japan	Oda et al. 2004
A. clarisquomosa	FJ375331	Yunnan, China	Direct submission
A. cylindrispora	AY325839	New Jersey, USA	Direct submission
A. excelsa	AY436453	Tuebingen, Germany	Zhang et al. 2004
A. gillbertii	AY325838	Landes, France	Direct submission
A. griseoturcosa	AB167728	Japan	Oda et al. 2004
A. kotohiraensis	FJ176722	Hunan, China	Zhang et al. 2010
A. manginiana	AY436463	Yunnan, China	Zhang et al. 2010
A. oberwinkelrana	FJ176724	Hunan, China	Zhang et al. 2010
A. arenaria	GQ925388	Australia	Justo et al. 2009
A. arenaria	GQ925393	Australia	Justo et al. 2009
A. rubescens	JF313654	Sweden	Direct submission
