# Supplementary material for: Multi-locus phylogeny of lethal amanitas: Implications for species diversity and historical biogeography
Source: BMC Evol Biol. 2014 Jun 21;14:143. doi: 10.1186/1471-2148-14-143 (PMC4094918; doi:10.1186/1471-2148-14-143)
Supplement: Additional file 3: Figure S1 — Phylogenetic trees inferred from the maximum likelihood (ML) analysis with branch support obtained by ML and BI analyses based on nrLSU, rpb2, ef1-α and β-tubulin sequences, respectively. [file 1471-2148-14-143-S3.pdf]

Figure S1 Phylogenetic trees inferred from maximum likelihood (ML) analysis with branch support obtained by ML and BI analyses based on nrLSU, *rpb2*, *ef1-α* and *β-tubulin*, respectively. Only maximum likelihood bootstraps (LB) over 70% and Bayesian posterior probabilities (PP) over 0.90 are reported on the branches. Thickened branches indicate LB between 90%-100% and PP between 0.95-1.0. Thickened branches with dots on the root nodes represent 100% LB and 1.0 PP.

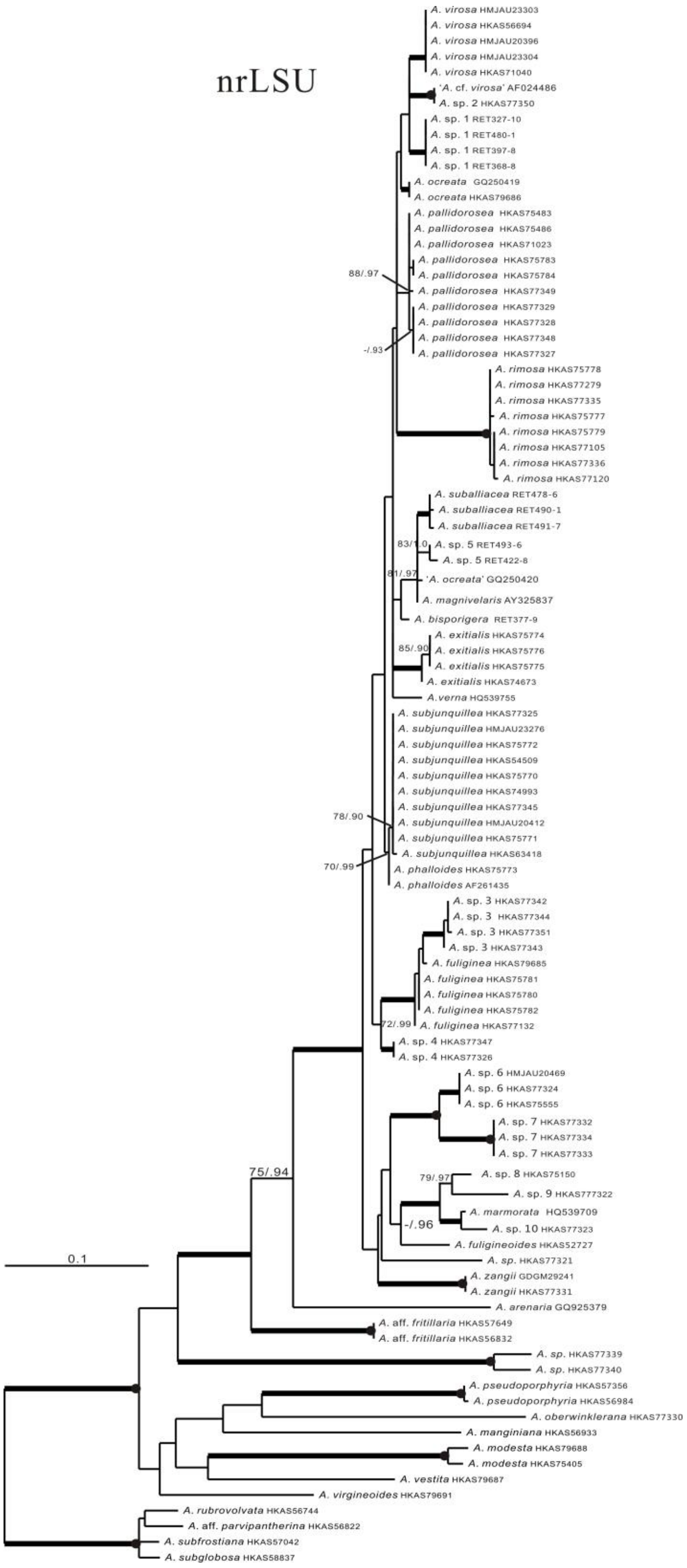

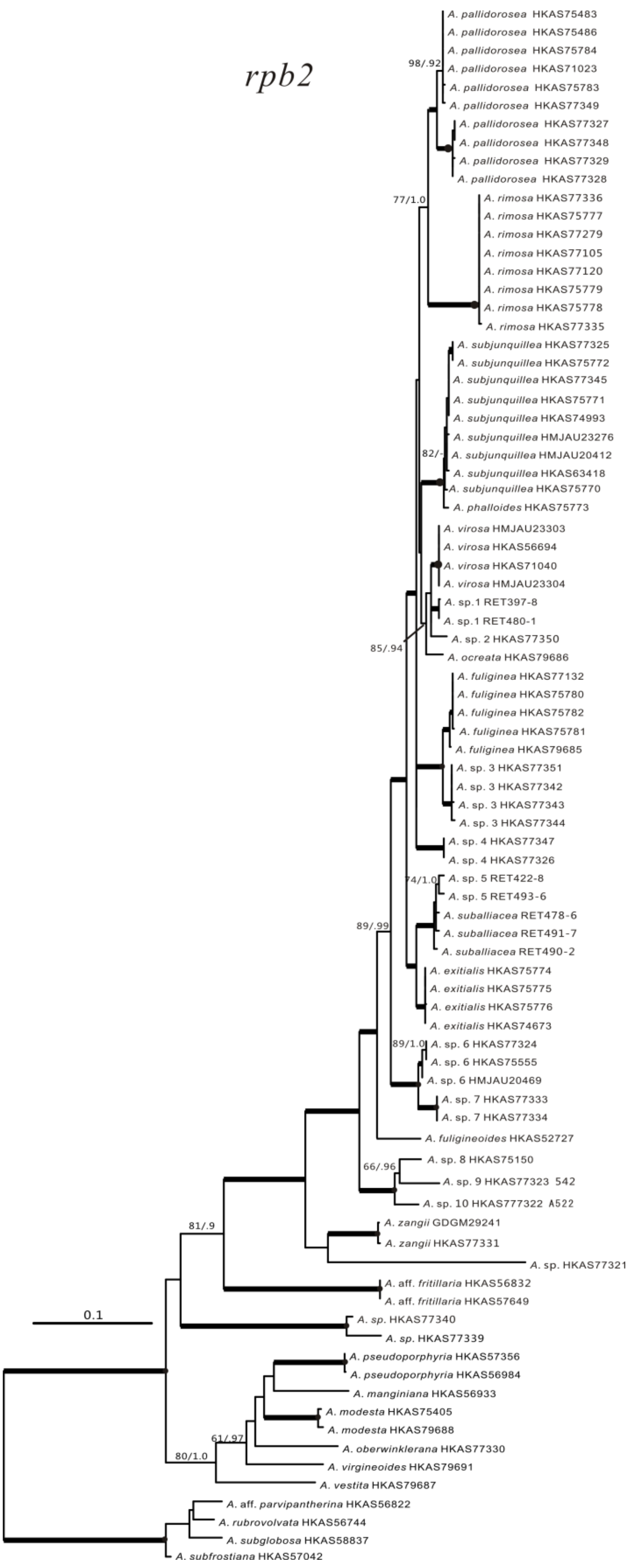
rpb2
A. pallidorosea HKAS75483
A. pallidorosea HKAS75486
A. pallidorosea HKAS75784
98/.92
A. pallidorosea HKAS71023
A. pallidorosea HKAS75783
A. pallidorosea HKAS77349
A. pallidorosea HKAS77327
A. pallidorosea HKAS77348
A. pallidorosea HKAS77329
A. pallidorosea HKAS77328
77/1.0
A. rimosa HKAS77336
A. rimosa HKAS75777
A. rimosa HKAS77279
A. rimosa HKAS77105
A. rimosa HKAS77120
A. rimosa HKAS75779
A. rimosa HKAS75778
A. rimosa HKAS77335
A. subjunquillea HKAS77325
A. subjunquillea HKAS75772
A. subjunquillea HKAS77345
A. subjunquillea HKAS75771
A. subjunquillea HKAS74993
A. subjunquillea HMJAU23276
82/-
A. subjunquillea HMJAU20412
A. subjunquillea HKAS63418
A. subjunquillea HKAS75770
A. phalloides HKAS75773
A. virosa HMJAU23303
A. virosa HKAS56694
A. virosa HKAS71040
A. virosa HMJAU23304
A. sp.1 RET397-8
A. sp.1 RET480-1
A. sp. 2 HKAS77350
85/.94
A. ocreata HKAS79686
A. fuliginea HKAS77132
A. fuliginea HKAS75780
A. fuliginea HKAS75782
A. fuliginea HKAS75781
A. fuliginea HKAS79685
A. sp. 3 HKAS77351
A. sp. 3 HKAS77342
A. sp. 3 HKAS77343
A. sp. 3 HKAS77344
A. sp. 4 HKAS77347
A. sp. 4 HKAS77326
74/1.0
A. sp. 5 RET422-8
A. sp. 5 RET493-6
A. suballiacea RET478-6
89/.99
A. suballiacea RET491-7
A. suballiacea RET490-2
A. exitialis HKAS75774
A. exitialis HKAS75775
A. exitialis HKAS75776
A. exitialis HKAS74673
89/1.0
A. sp. 6 HKAS77324
A. sp. 6 HKAS75555
A. sp. 6 HMJAU20469
A. sp. 7 HKAS77333
A. sp. 7 HKAS77334
A. fuligineoides HKAS52727
66/.96
A. sp. 8 HKAS75150
A. sp. 9 HKAS77323 542
A. sp. 10 HKAS777322 A522
81/.9
A. zangii GDGM29241
A. zangii HKAS77331
A. sp. HKAS77321
A. aff. fritillaria HKAS56832
A. aff. fritillaria HKAS57649
0.1
A. sp. HKAS77340
A. sp. HKAS77339
A. pseudoporphyria HKAS57356
A. pseudoporphyria HKAS56984
A. manginiana HKAS56933
A. modesta HKAS75405
A. modesta HKAS79688
61/.97
A. oberwinklerana HKAS77330
80/1.0
A. virgineoides HKAS79691
A. vestita HKAS79687
A. aff. parvipantherina HKAS56822
A. rubrovolvata HKAS56744
A. subglobosa HKAS58837
A. subfrostiana HKAS57042

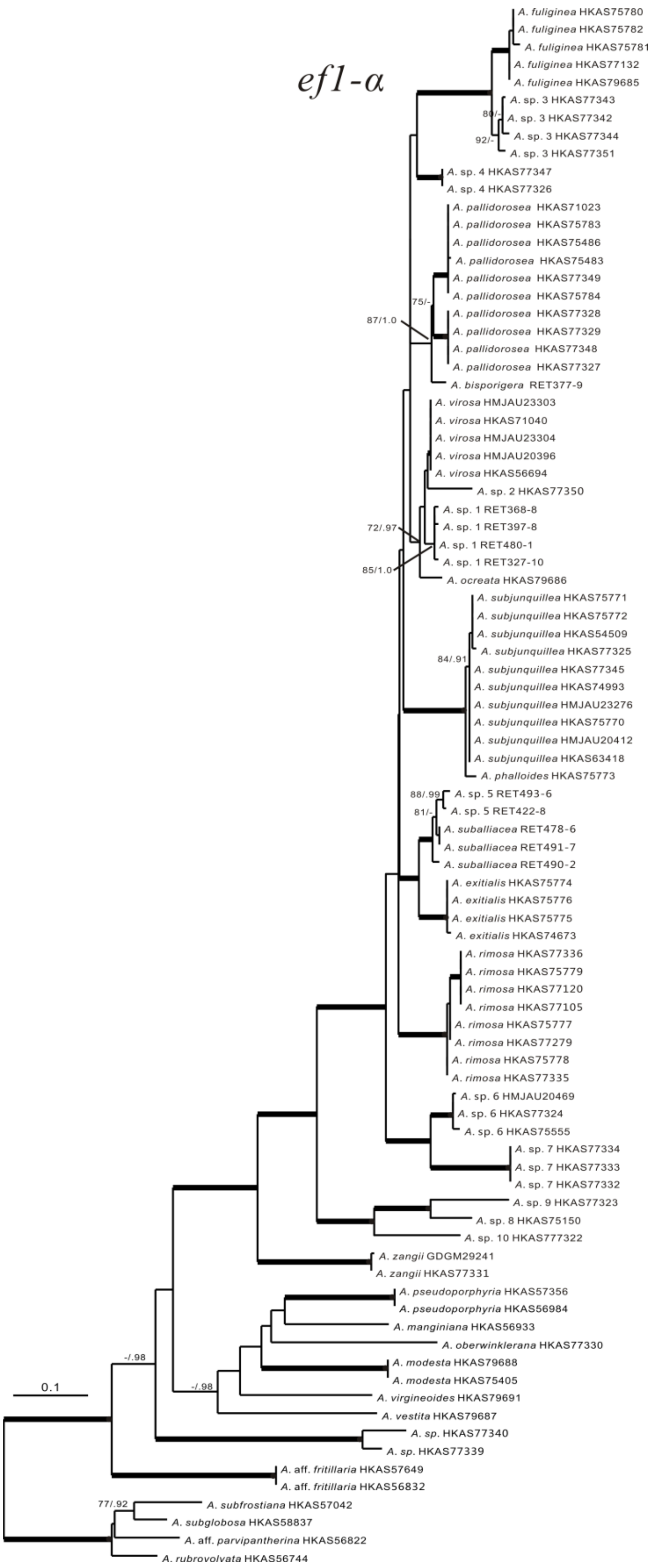
ef1-α
A. fuliginea HKAS75780
A. fuliginea HKAS75782
A. fuliginea HKAS75781
A. fuliginea HKAS77132
A. fuliginea HKAS79685
A. sp. 3 HKAS77343
80/-
A. sp. 3 HKAS77342
92/-
A. sp. 3 HKAS77344
A. sp. 3 HKAS77351
A. sp. 4 HKAS77347
A. sp. 4 HKAS77326
A. pallidorosea HKAS71023
A. pallidorosea HKAS75783
A. pallidorosea HKAS75486
A. pallidorosea HKAS75483
A. pallidorosea HKAS77349
75/-
A. pallidorosea HKAS75784
87/1.0
A. pallidorosea HKAS77328
A. pallidorosea HKAS77329
A. pallidorosea HKAS77348
A. pallidorosea HKAS77327
A. bisporigera RET377-9
A. virosa HMJAU23303
A. virosa HKAS71040
A. virosa HMJAU23304
A. virosa HMJAU20396
A. virosa HKAS56694
A. sp. 2 HKAS77350
A. sp. 1 RET368-8
72/.97
A. sp. 1 RET397-8
A. sp. 1 RET480-1
85/1.0
A. sp. 1 RET327-10
A. ocreata HKAS79686
A. subjunquillea HKAS75771
A. subjunquillea HKAS75772
A. subjunquillea HKAS54509
A. subjunquillea HKAS77325
84/.91
A. subjunquillea HKAS77345
A. subjunquillea HKAS74993
A. subjunquillea HMJAU23276
A. subjunquillea HKAS75770
A. subjunquillea HMJAU20412
A. subjunquillea HKAS63418
A. phalloides HKAS75773
88/.99
A. sp. 5 RET493-6
81/-
A. sp. 5 RET422-8
A. suballiacea RET478-6
A. suballiacea RET491-7
A. suballiacea RET490-2
A. exitialis HKAS75774
A. exitialis HKAS75776
A. exitialis HKAS75775
A. exitialis HKAS74673
A. rimosa HKAS77336
A. rimosa HKAS75779
A. rimosa HKAS77120
A. rimosa HKAS77105
A. rimosa HKAS75777
A. rimosa HKAS77279
A. rimosa HKAS75778
A. rimosa HKAS77335
A. sp. 6 HMJAU20469
A. sp. 6 HKAS77324
A. sp. 6 HKAS75555
A. sp. 7 HKAS77334
A. sp. 7 HKAS77333
A. sp. 7 HKAS77332
A. sp. 9 HKAS77323
A. sp. 8 HKAS75150
A. sp. 10 HKAS777322
A. zangii GDGM29241
A. zangii HKAS77331
A. pseudoporphyria HKAS57356
A. pseudoporphyria HKAS56984
A. manginiana HKAS56933
A. oberwinklerana HKAS77330
A. modesta HKAS79688
A. modesta HKAS75405
-/.98
-/.98
0.1
A. virgineoides HKAS79691
A. vestita HKAS79687
A. sp. HKAS77340
A. sp. HKAS77339
A. aff. fritillaria HKAS57649
A. aff. fritillaria HKAS56832
77/.92
A. subfrostiana HKAS57042
A. subglobosa HKAS58837
A. aff. parvipantherina HKAS56822
A. rubrovolvata HKAS56744

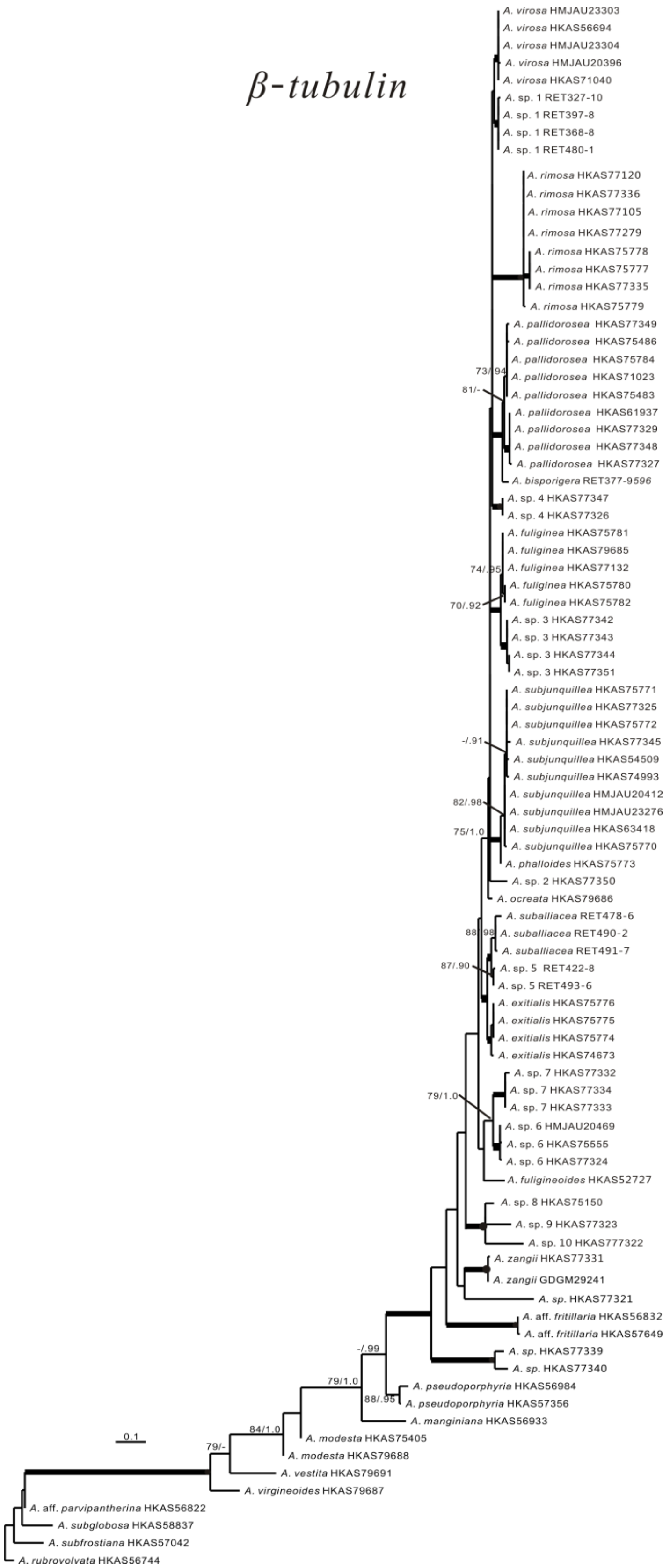
β-tubulin
A. virosa HMJAU23303
A. virosa HKAS56694
A. virosa HMJAU23304
A. virosa HMJAU20396
A. virosa HKAS71040
A. sp. 1 RET327-10
A. sp. 1 RET397-8
A. sp. 1 RET368-8
A. sp. 1 RET480-1
A. rimosa HKAS77120
A. rimosa HKAS77336
A. rimosa HKAS77105
A. rimosa HKAS77279
A. rimosa HKAS75778
A. rimosa HKAS75777
A. rimosa HKAS77335
A. rimosa HKAS75779
A. pallidorosea HKAS77349
A. pallidorosea HKAS75486
A. pallidorosea HKAS75784
73/.94
A. pallidorosea HKAS71023
81/-
A. pallidorosea HKAS75483
A. pallidorosea HKAS61937
A. pallidorosea HKAS77329
A. pallidorosea HKAS77348
A. pallidorosea HKAS77327
A. bisporigera RET377-9596
A. sp. 4 HKAS77347
A. sp. 4 HKAS77326
A. fuliginea HKAS75781
A. fuliginea HKAS79685
74/.95
A. fuliginea HKAS77132
A. fuliginea HKAS75780
70/.92
A. fuliginea HKAS75782
A. sp. 3 HKAS77342
A. sp. 3 HKAS77343
A. sp. 3 HKAS77344
A. sp. 3 HKAS77351
A. subjunquillea HKAS75771
A. subjunquillea HKAS77325
A. subjunquillea HKAS75772
-/.91
A. subjunquillea HKAS77345
A. subjunquillea HKAS54509
A. subjunquillea HKAS74993
A. subjunquillea HMJAU20412
82/.98
A. subjunquillea HMJAU23276
A. subjunquillea HKAS63418
75/1.0
A. subjunquillea HKAS75770
A. phalloides HKAS75773
A. sp. 2 HKAS77350
A. ocreata HKAS79686
A. suballiacea RET478-6
88/.98
A. suballiacea RET490-2
A. suballiacea RET491-7
87/.90
A. sp. 5 RET422-8
A. sp. 5 RET493-6
A. exitialis HKAS75776
A. exitialis HKAS75775
A. exitialis HKAS75774
A. exitialis HKAS74673
A. sp. 7 HKAS77332
A. sp. 7 HKAS77334
79/1.0
A. sp. 7 HKAS77333
A. sp. 6 HMJAU20469
A. sp. 6 HKAS75555
A. sp. 6 HKAS77324
A. fuligineoides HKAS52727
A. sp. 8 HKAS75150
A. sp. 9 HKAS77323
A. sp. 10 HKAS777322
A. zangii HKAS77331
A. zangii GDGM29241
A. sp. HKAS77321
A. aff. fritillaria HKAS56832
A. aff. fritillaria HKAS57649
A. sp. HKAS77339
A. sp. HKAS77340
-/.99
79/1.0
A. pseudoporphyria HKAS56984
88/.95
A. pseudoporphyria HKAS57356
A. manginiana HKAS56933
84/1.0
A. modesta HKAS75405
0.1
79/-
A. modesta HKAS79688
A. vestita HKAS79691
A. virgineoides HKAS79687
A. aff. parvipantherina HKAS56822
A. subglobosa HKAS58837
A. subfrostiana HKAS57042
A. rubrovolvata HKAS56744
