# Supplementary material for: Multi-locus phylogeny of lethal amanitas: Implications for species diversity and historical biogeography
Source: BMC Evol Biol. 2014 Jun 21;14:143. doi: 10.1186/1471-2148-14-143 (PMC4094918; doi:10.1186/1471-2148-14-143)
Supplement: Additional file 4: Table S3 — GenBank accession numbers of the sequences used in the divergence time estimation. [file 1471-2148-14-143-S4.pdf]

Table S3 GenBank accession numbers of the sequences used in the divergence time estimation.

Taxon	Voucher	Accession numbers		
		nrLSU	*rpb2*	*ef1-α*
Agaricostilbum hyphaenes	CBS7811	AY634278	AY780933	AY879114
Amanita muscaria	GAL2810	DQ060884	---	EU071872
Boletellus projectellus	MB03-118	AY684158	AY787218	AY879116
Calocera cornea	GEL5359	AY701526	AY536286	AY881019
Clavaria zollingeri	TENN58652	AY639882	AY780940	AY881024
Coltricia perennis	DSH93-198	AF287854	AY218526	AY885147
Cryptococcus humicola	PYCC 3387T	DQ645514	DQ645517	DQ645519
Dacryopinax spathularia	GEL5052	AY701525	AY786054	AY881020
Fomitiporia mediterranea	Michael Fischer 3/22	AY684157	AY803748	AY885149
Fomitopsis pinicola	MB03-036	AY684164	AY786056	AY885152
Gautieria otthii	REG 636	AF393058	AY218486	AY883434
Hygrocybe conica	PBM 918	AY684167	AY803747	AY883425
Hygrophoropsis aurantiaca	MB03-127	AY684156	AY786059	AY883427
Lactarius deceptivus	PBM2462	AY631899	AY803749	AY885158
Marasmius alliaceus	TENN55620	AY635776	AY786060	AY883431
Neurospora crassa	MUCL 19026	AF286411	XM_952013	XM_959775
Pluteus romellii	ECV 3201	AY634279	AY786063	AY883433
Ramaria rubella	PBM 2408	AY645057	AY786064	AY883435
Rhizopus stolonifer	DAOM225708	DQ273817	AFTOL database	AFTOL database
Schizosaccharomyces pombe	Strain972	Z19136	NM_00101849	NM_001022750
Tricholomopsis decora	--	AY691888	DQ408112	DQ029195
Ustilago maydis	PBM2469	AF453938	AY485636	AY885160
