# Supplementary figures and images for: Multi-locus phylogeny of lethal amanitas: Implications for species diversity and historical biogeography
Source: BMC Evol Biol. 2014 Jun 21;14:143. doi: 10.1186/1471-2148-14-143 (PMC4094918; doi:10.1186/1471-2148-14-143)

**Figure S2 Results of the HPLC analyses.** The loading amount of the un-standard samples are 20  $\mu$ l.

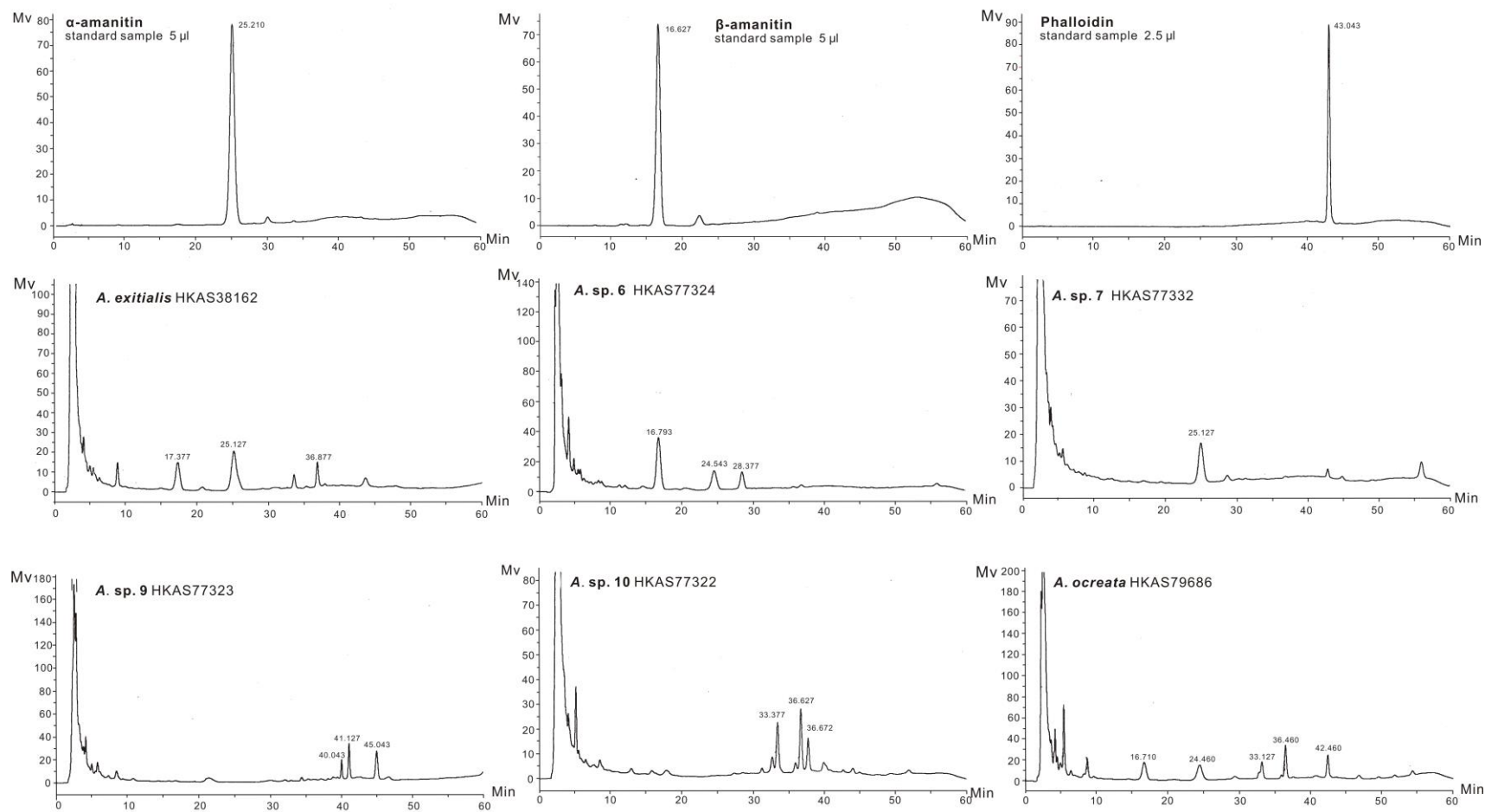

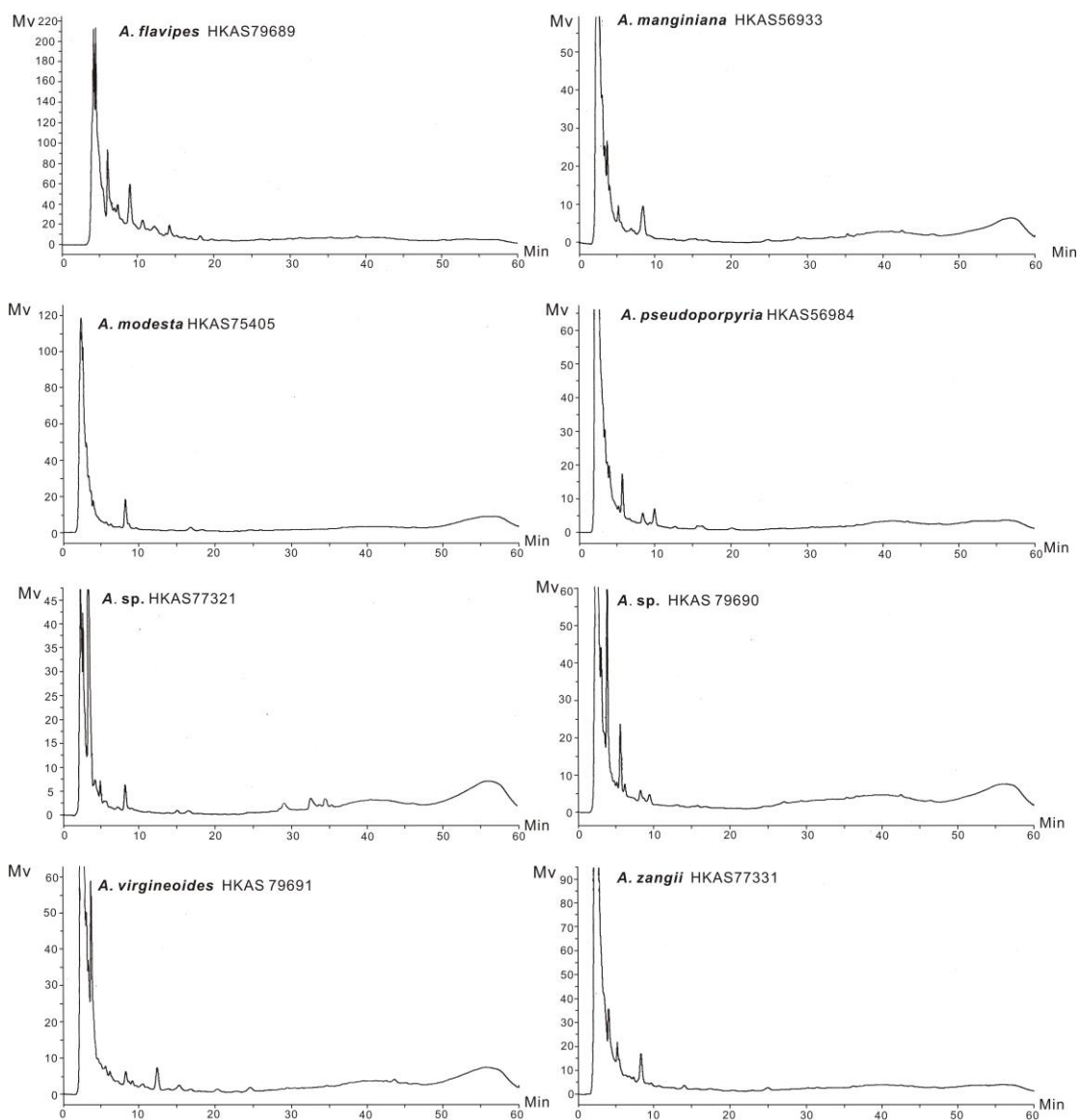

Supplement: Additional file 6: Figure S2 — Results of the HPLC analyses. [file 1471-2148-14-143-S6.pdf]
