# Supplementary material for: Multi-locus phylogeny of lethal amanitas: Implications for species diversity and historical biogeography
Source: BMC Evol Biol. 2014 Jun 21;14:143. doi: 10.1186/1471-2148-14-143 (PMC4094918; doi:10.1186/1471-2148-14-143)
Supplement: Additional file 7: Figure S3 — Chronogram and estimated divergence times of lethal amanitas generated from the molecular clock analysis using the combined (nrLSU/rpb2/ ef1-α) dataset. [file 1471-2148-14-143-S7.pdf]

Figure S3 Chronogram and estimated divergence times of *Amanita* generated from molecular clock analysis using the combined (nrLSU/*rpb2*/*ef1*-α) dataset. The chronogram is obtained by using the Ascomycota–Basidiomycota divergence time of 582 Mya as the calibration point. The calibration point and objects of this study are marked in the chronogram. Estimated divergence times of main nodes are summarized in the table at the upper-left corner.

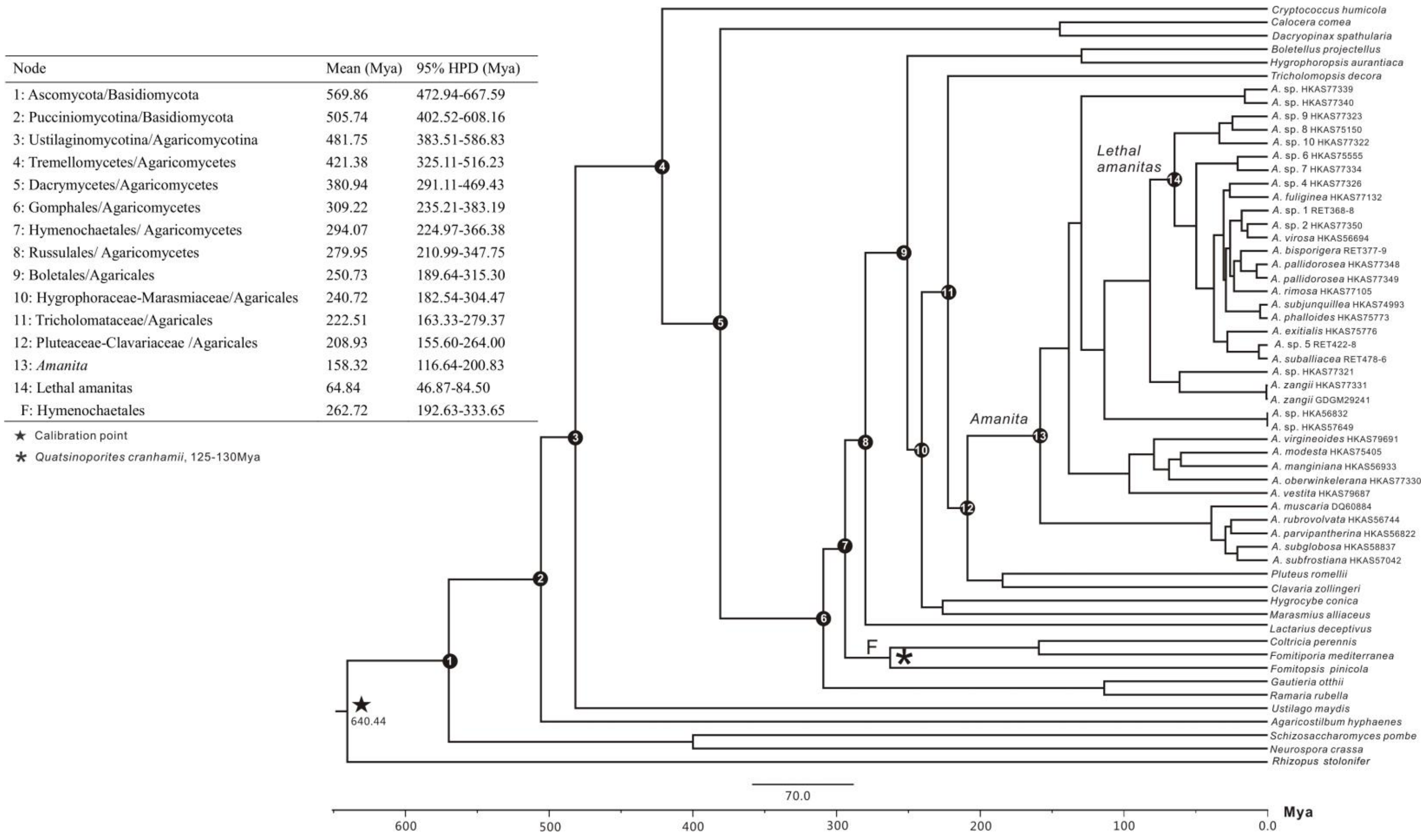

Node	Mean (Mya)	95% HPD (Mya)
1: Ascomycota/Basidiomycota	569.86	472.94-667.59
2: Pucciniomycotina/Basidiomycota	505.74	402.52-608.16
3: Ustilaginomycotina/Agaricomycotina	481.75	383.51-586.83
4: Tremellomycetes/Agaricomycetes	421.38	325.11-516.23
5: Dacrymycetes/Agaricomycetes	380.94	291.11-469.43
6: Gomphales/Agaricomycetes	309.22	235.21-383.19
7: Hymenochaetales/ Agaricomycetes	294.07	224.97-366.38
8: Russulales/ Agaricomycetes	279.95	210.99-347.75
9: Boletales/Agaricales	250.73	189.64-315.30
10: Hygrophoraceae-Marasmiaceae/Agaricales	240.72	182.54-304.47
11: Tricholomataceae/Agaricales	222.51	163.33-279.37
12: Pluteaceae-Clavariaceae /Agaricales	208.93	155.60-264.00
13: *Amanita*	158.32	116.64-200.83
14: Lethal amanitas	64.84	46.87-84.50
F: Hymenochaetales	262.72	192.63-333.65
